# Integrated Proteomics to Understand the Role of Neuritin (NRN1) as a Mediator of Cognitive Resilience to Alzheimer’s Disease

**DOI:** 10.1016/j.mcpro.2023.100542

**Published:** 2023-04-05

**Authors:** Cheyenne Hurst, Derian A. Pugh, Measho H. Abreha, Duc M. Duong, Eric B. Dammer, David A. Bennett, Jeremy H. Herskowitz, Nicholas T. Seyfried

**Affiliations:** 1 Department of Biochemistry, Emory School of Medicine, Emory Goizueta Alzheimer’s Disease Research Center, Atlanta, Georgia, USA; 2 Department of Neurology, Center for Neurodegeneration and Experimental Therapeutics, University of Alabama at Birmingham School of Medicine, Birmingham, Alabama, USA; 3 Rush Alzheimer’s Disease Center, Rush University Medical Center, Chicago, Illinois, USA

**Keywords:** Alzheimer’s disease, cognition, amyloid, synapse, proteomics, aging

## Abstract

The molecular mechanisms and pathways enabling certain individuals to remain cognitively normal despite high levels of Alzheimer’s disease (AD) pathology remain incompletely understood. These cognitively normal people with AD pathology are described as preclinical or asymptomatic AD (AsymAD) and appear to exhibit cognitive resilience to the clinical manifestations of AD dementia. Here we present a comprehensive network-based approach from cases clinically and pathologically defined as asymptomatic AD to map resilience-associated pathways and extend mechanistic validation. Multiplex tandem mass tag MS (TMT-MS) proteomic data (n = 7787 proteins) was generated on brain tissue from Brodmann area 6 and Brodmann area 37 (n = 109 cases, n = 218 total samples) and evaluated by consensus weighted gene correlation network analysis. Notably, neuritin (NRN1), a neurotrophic factor previously linked to cognitive resilience, was identified as a hub protein in a module associated with synaptic biology. To validate the function of NRN1 with regard to the neurobiology of AD, we conducted microscopy and physiology experiments in a cellular model of AD. NRN1 provided dendritic spine resilience against amyloid-β (Aβ) and blocked Aβ-induced neuronal hyperexcitability in cultured neurons. To better understand the molecular mechanisms of resilience to Aβ provided by NRN1, we assessed how exogenous NRN1 alters the proteome by TMT-MS (n = 8238 proteins) of cultured neurons and integrated the results with the AD brain network. This revealed overlapping synapse-related biology that linked NRN1-induced changes in cultured neurons with human pathways associated with cognitive resilience. Collectively, this highlights the utility of integrating the proteome from the human brain and model systems to advance our understanding of resilience-promoting mechanisms and prioritize therapeutic targets that mediate resilience to AD.

## Highlights

• Multiplex tandem mass tag mass spectrometry on the human frontal and temporal cortex.
• Computational analysis links NRN1 with cognitive resilience to Alzheimer’s disease.
• NRN1 facilitated dendritic spine resilience against amyloid-β in cultured neurons.
• NRN1 induced proteome changes in neurons associated with cognitive resilience.

## Graphical Abstract

**Figure:**
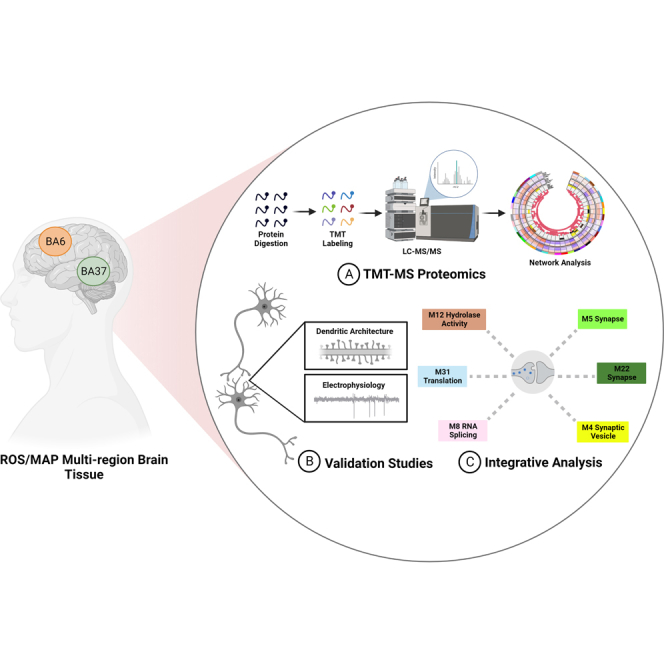

## In Brief

Mechanisms by which individuals with Alzheimer’s disease (AD) pathology avoid dementia are not well known. We employed multiplex tandem mass tag mass spectrometry (TMT-MS)–based proteomics to identify proteins linked with cognitive resilience to AD. Computational strategies identified NRN1 as a synaptic protein associated with resilience. Using models of AD, we demonstrated that NRN1 can facilitate dendritic spine resilience against amyloid-β (Aβ) and block Aβ-induced neuronal hyperexcitability. We assessed how NRN1 alters the proteome by TMT-MS, which revealed overlapping synapse-related biology with humans.

As the aging population continues to expand, the public health burden of Alzheimer’s disease (AD) is projected to reach staggering numbers without the advent of effective disease-altering therapies (1). AD is an irreversible neurodegenerative disease defined by its pathological hallmarks, amyloid-beta (Aβ) plaques, and tau neurofibrillary tangles (NFTs) (2). Functional imaging and biomarker studies suggest AD pathological brain changes could initiate up to 2 decades before symptom onset, indicating a protracted prodromal disease phase ideal for early intervention (3). Importantly, many older individuals without dementia or mild cognitive impairment meet the pathologic criteria for AD. Approximately one-third of individuals harbor high levels of AD and related disease pathology in their brains at autopsy but showed little to no signs of cognitive impairment in their lifetime (4, 5). These cognitively normal people with AD pathology are described as preclinical or asymptomatic AD (AsymAD) and appear to exhibit cognitive resilience to the clinical manifestations of AD dementia. One working hypothesis is that such individuals possess physiological resilience that confers the ability to maintain cognitive function despite the accumulation of AD-related pathologies (6, 7, 8). Identifying the specific mechanisms by which older individuals with AD pathology avoid dementia onset is one of the most pivotal, unanswered questions in the field.

Cognitive impairment in AD is the result of lost neuronal connectivity in brain regions critical to memory and other cognitive processes. For a cognitive impairment to develop, there must be loss or dysfunction of the neural elements that subserve cognition, for example, neurons, synapses, and dendritic spines. Our work and that of others demonstrate the preservation of neuron numbers and synaptic markers as well as enhanced dendritic spine remodeling in resilient cases (9, 10, 11). Together this implies that the ability to maintain cognitive function in an environment of AD pathology is linked to the preservation and maintenance of synapses or spines. These findings raise important questions: (1) What are the molecular pathways that drive the preservation of synaptic connections and maintenance of cognitive abilities in resilient individuals? (2) How can we identify protein targets to exploit these mechanisms for therapies in at-risk patients?

Previous efforts to address these gaps in knowledge using proteomics have focused primarily on a single brain region, the dorsolateral prefrontal cortex (12, 13, 14). Individual proteins and protein communities have been reported to have associations with cognitive resilience, but it is not clear whether these proteome changes are exclusive to the brain region that was analyzed. The AD pathology is present across numerous cortical and subcortical regions in the brain (15, 16); therefore, we hypothesized that proteins contributing to physiological resilience would likely act across more than one brain region. In the present study, we implemented a systems-level multiregion network analysis of human postmortem brain tissue derived from the Religious Order and Rush Memory and Aging Project (ROSMAP). ROSMAP is an information-rich longitudinal cohort-based study in which participants enroll without dementia, undergo annual cognitive and clinical assessments, and donate their brains at death (17). Matched brain tissue from two brain regions was analyzed *via* multiplex tandem mass tag MS (TMT-MS)–based proteomics followed by consensus weighted gene correlation network analysis (cWGCNA). Brodmann area 6 (BA6) and Brodmann area 37 (BA37) were selected for their pathologically distinct features, where BA6 (frontal cortex) has predominant amyloid pathology and BA37 (temporal cortex) exhibits prominent tau-related pathology. We prioritized modules or communities of proteins that were enriched with markers of cognitive resilience identified from an independent brain proteome-wide association study (PWAS) of cognitive trajectory (14). This revealed proteins linked to synaptic biology and cellular energetics. Notably, neuritin (NRN1) was identified as a hub protein that co-expressed with other synaptic proteins that remained increased in AsymAD compared to symptomatic AD cases. Identification of NRN1 and its relationship with resilience corroborates previous results of increased NRN1 abundance in cognitive stability and thus suggests NRN1 as an attractive potential resilience-promoting target (12, 14). To validate our systems-level analysis, primary neuronal culture was used to evaluate the neuroprotective mechanisms of NRN1. The current work provides a critical replication of previous findings and contributes novel, in-depth characterization of proteins and mechanisms influencing resilience across two distinct brain regions.

## Experimental Procedures

### Experimental Design and Statistical Rationale

#### Human Proteomics

Matched postmortem brain tissues from 109 ROSMAP cases from two brain regions (BA6 and BA37, n = 218 total samples) were used for the human proteomic analysis. Cases were included according to a previously established and peer-reviewed strategy (12, 18). In brief, cases with Consortium to Establish a Registry for Alzheimer’s Disease (CERAD) scores of 0 to 1 and Braak scores of 0 to 3 without dementia at the last evaluation were defined as control (if Braak equals 3, then CERAD must equal 0; BA6: n = 24, BA37: n = 24); cases with CERAD scores 1 to 3 and Braak scores 3 to 6 without dementia at last evaluation were defined as AsymAD (BA6: n = 53, BA37: n = 52); cases with CERAD 2 to 3 and Braak 3 to 6 with dementia at last evaluation were defined as AD (BA6: n = 32, BA37: n = 33). Dementia was defined by Mini-Mental State Examination (MMSE) scores <24 (19). Group comparisons in human brain samples were performed with one-way ANOVA with Holm post hoc correction of all comparisons.

#### Rat Proteomics

Primary neurons for proteomic analysis were incubated with recombinant NRN1 (n = 3 technical replicates) or vehicle treatment (n = 4 technical replicates). Differential expression between NRN1 and vehicle-treated neurons was determined by Student’s *t* test and corrected for multiple hypothesis testing by Reproducibility-Optimized Test Statistic (ROTS) false discovery rate (FDR) correction (20). A one-tailed Fisher exact was used to identify significant overrepresentation or overlap of rat differentially expressed proteins with human network modules, and *P*values were corrected for multiple testing using the Benjamini–Hochberg method.

### Chemicals and Reagents

For primary neuron experiments, Aβ_42_ oligomers were purchased from Bachem and prepared as previously described (21). The Aβ_42_ was resuspended in 1X Hanks’ balanced salt solution (c) and Dimethyl sulfoxide and then placed at 4 °C overnight. Recombinant human Neuritin protein (Abcam, ab69755) was reconstituted in water to a concentration of 0.1 mg/ml. For the Thioflavin T (ThT) aggregation assay, recombinant human Aβ_42_ (5 μM) (rPeptide, # A-1170-1) was handled essentially as described (22) and detailed below. Plasmid encoding Lifeact-GFP was a generous gift from Dr Gary Bassell, Emory University School of Medicine, Atlanta, GA, USA.

### Human Postmortem Brain Tissue and Case Classification

Paired brain tissue samples from the frontal cortex (Brodmann area 6, BA6) and temporal cortex (Brodmann area 37, BA37) were obtained from the ROSMAP (n = 256 total samples) in accordance with proper Institutional Review Board protocols of the home institution. Postmortem neuropathological evaluation of neuritic plaque distribution was performed according to the CERAD criteria (23) and the extent of neurofibrillary tangle pathology was assessed with the Braak staging system (15). Case classification was determined according to a previously established and peer-reviewed strategy (12, 18). In brief, cases with CERAD scores of 0 to 1 and Braak scores of 0 to 3 without dementia at the last evaluation were defined as control (if Braak equals 3, then CERAD must equal 0; BA6: n = 24, BA37: n = 24); cases with CERAD scores 1 to 3 and Braak scores 3 to 6 without dementia at last evaluation were defined as AsymAD (BA6: n = 53, BA37: n = 52); and cases with CERAD 2 to 3 and Braak 3 to 6 with dementia at last evaluation were defined as AD (BA6: n = 32, BA37: n = 33). Dementia was defined by MMSE scores <24 (19).

### Brain Tissue Homogenization

Sample homogenization was performed as previously described (12). Approximately 100 mg (wet tissue weight) of brain tissue was homogenized in 8 M urea lysis buffer (8 M urea, 10 mM Tris, 100 mM NaH_2_PO_4_, pH 8.5) with HALT protease and phosphatase inhibitor cocktail (Thermo Fisher Scientific) using a Bullet Blender (Next Advance). Each RINO sample tube (Next Advance) was supplemented with ∼100 μl of stainless-steel beads (0.9–2.0 mm blend, Next Advance) and 500 μl of lysis buffer. Tissues were added immediately after excision and homogenized with Bullet Blender at 4 °C with two full 5-min cycles. The lysates were transferred to new Eppendorf LoBind tubes and sonicated for three cycles consisting of 5 s of active sonication at 30% amplitude, followed by 15 s on ice. Samples were then centrifuged for 5 min at 15,000*g* and the supernatant was transferred to a new tube. Protein concentration was determined by bicinchoninic acid assay (Pierce), and one-dimensional SDS-PAGE gels were run followed by Coomassie blue staining as quality control for protein integrity and equal loading before proceeding to protein digestion.

### Brain Protein Digestion

For protein digestion (as described (12, 24, 25)), 100 μg of each sample was aliquoted, and volumes were normalized with additional lysis buffer. Samples were reduced with 1 mM dithiothreitol at room temperature for 30 min, followed by 5 mM iodoacetamide alkylation in the dark for another 30 min. Lysyl endopeptidase (Wako) at 1:100 (wt/wt) was added, and digestion was allowed to proceed overnight. Samples were then sevenfold diluted with 50 mM ammonium bicarbonate. Trypsin (Promega) was added at 1:50 (wt/wt), and digestion was carried out for another 16 h. The peptide solutions were acidified to a final concentration of 1% (vol/vol) formic acid (FA) and 0.1% (vol/vol) trifluoroacetic acid (TFA) and desalted with a 30-mg HLB column (Oasis). Each HLB column was first rinsed with 1 ml of methanol, washed with 1 ml of 50% (vol/vol) acetonitrile (ACN), and equilibrated with 2 × 1 ml of 0.1% (vol/vol) TFA. The samples were then loaded onto the column and washed with 2 × 1 ml of 0.1% (vol/vol) TFA. Elution was performed with 2 volumes of 0.5 ml of 50% (vol/vol) ACN. An equal amount of peptide from each sample was aliquoted and pooled as the pooled global internal standard (GIS), which was split and labeled in each TMT batch as described below. The eluates were then dried to completeness using a SpeedVac.

### TMT Peptide Labeling for the Brain Proteome

Before TMT labeling, cases were randomized by covariates (age, sex, post-mortem interval [PMI], diagnosis, etc.) into the 26 total batches. Peptides from each individual case and the GIS pooled standard or bridging sample (at least one per batch) were labeled using the TMT 11-plex kit (ThermoFisher 90,406). Labeling was performed as described (12, 24, 26, 27). In each batch, up to two TMT channels were used to label GIS standards, and the remaining TMT channels were reserved for individual samples after randomization. In brief, each sample (containing 100 μg of peptides) was resuspended in 100 mM TEAB buffer (100 μl). The TMT labeling reagents (5 mg) were equilibrated to room temperature, and anhydrous ACN (256 μl) was added to each reagent channel. Each channel was gently vortexed for 5 min, and then 41 μl from each TMT channel was transferred to the peptide solutions and allowed to incubate for 1 h at room temperature. The reaction was quenched with 5% (vol/vol) hydroxylamine (8 μl) (Pierce). All channels were then combined and dried by SpeedVac (Labconco) to approximately 150 μl and diluted with 1 ml of 0.1% (vol/vol) TFA and then acidified to a final concentration of 1% (vol/vol) FA and 0.1% (vol/vol) TFA. Labeled peptides were desalted with a 200-mg C18 Sep-Pak column (Waters). Each Sep-Pak column was activated with 3 ml of methanol, washed with 3 ml of 50% (vol/vol) can, and equilibrated with 2 × 3 ml of 0.1% TFA. The samples were then loaded, and each column was washed with 2 × 3 ml of 0.1% (vol/vol) TFA, followed by 2 ml of 1% (vol/vol) FA. Elution was performed with 2 volumes of 1.5-ml 50% (vol/vol) ACN. The eluates were then dried to completeness using a SpeedVac.

### High-pH Offline Fractionation for the Brain Proteome

Fractionation was conducted as described (12, 26, 28). Dried samples were resuspended in a high-pH loading buffer (0.07% v/v NH4OH, 0.045% v/v FA, 2% v/v ACN) and loaded onto an Agilent ZORBAX 300 Extend-C18 column (2.1 mm × 150 mm with 3.5 um beads). An Agilent 1100 HPLC system was used to carry out the fractionation. Solvent A consisted of 0.0175% (vol/vol) NH4OH, 0.01125% (vol/vol) FA, and 2% (vol/vol) ACN; solvent B consisted of 0.0175% (vol/vol) NH4OH, 0.01125% (vol/vol) FA, and 90% (vol/vol) ACN. The sample elution was performed over a 58.6 min gradient with a flow rate of 0.4 ml/min. The gradient consisted of 100% solvent A for 2 min, then 0% to 12% solvent B over 6 min, then 12% to 40% over 28 min, then 40% to 44% over 4 min, then 44% to 60% over 5 min, and then held constant at 60% solvent B for 13.6 min. A total of 96 individual equal-volume fractions were collected across the gradient and subsequently pooled by concatenation (28) into 24 fractions and dried to completeness using a SpeedVac.

### LC-MS/MS for the Brain Proteome

All fractions were resuspended in an equal volume of loading buffer (0.1% FA, 0.03% TFA, 1% ACN) and analyzed by LC-MS/MS essentially as described (12, 29) Peptide eluents were separated on a self-packed C18 (1.9 μm, Dr Maisch) fused silica column (25 cm × 75 μM internal diameter, New Objective) by a Dionex UltiMate 3000 RSLCnano liquid chromatography system (Thermo Fisher Scientific) for the ROSMAP samples. Peptides were monitored on an Orbitrap Fusion mass spectrometer (Thermo Fisher Scientific). Sample elution was performed over a 120-min gradient with a flow rate of 300 nl min^−1^ with buffer B ranging from 1% to 50% (buffer A: 0.1% FA in water; buffer B: 0.1% FA in 80% ACN). The mass spectrometer was set to acquire in data-dependent mode using the top-speed workflow with a cycle time of 3 s. Each cycle consisted of one full scan followed by as many MS/MS (MS2) scans that could fit within the time window. Full MS scans were collected at a resolution of 120,000 (400–1400 *m*/*z* range, 4 × 10^5^ AGC, 50-ms maximum ion injection time). All higher energy collision-induced dissociation (HCD) MS/MS spectra were acquired at a resolution of 60,000 (1.6 *m*/*z* isolation width, 35% collision energy, 5 × 10^4^AGC target, 50-ms maximum ion time). °ynamic exclusion was set to exclude previously sequenced peaks for 20 s within a 10-ppm isolation window.

### Database Searching and Protein Quantification for the Brain Proteome

All raw MS data files (624 total RAW files generated across 26 batches) were analyzed in the Proteome Discover software suite (version 2.3, ThermoFisher), and MS/MS spectra were searched against the UniProtKB human proteome database (downloaded April 2015 with 90,411 total sequences). The Sequest HT search engine was used with the following parameters: fully tryptic specificity; maximum of two missed cleavages; minimum peptide length of 6; fixed modifications for TMT tags on lysine residues and peptide N-termini (+229.162932 Da) and carbamidomethylation of cysteine residues (+57.02146 Da); variable modification for oxidation of methionine residues (+15.99492 Da) and deamidation of asparagine and glutamine (+0.984 Da); precursor mass tolerance of 20 ppm; and fragment mass tolerance of 0.05 Da. Peptide spectral matches (PSMs) were filtered to an FDR of <1% using the Percolator node. Following spectral alignment, peptides were assembled into proteins and further filtered based on the combined probabilities of their constituent peptides to a final FDR of 1%. Multi-consensus was performed to achieve parsimony across individual batches. In cases of redundancy, shared peptides were assigned to the protein sequence in adherence with the principles of parsimony. Reporter ions were quantified from MS2 scans using an integration tolerance of 20 ppm with the most confident centroid setting. Only PSMs with <50% isolation interference were used for quantification, and only unique and razor (*i.e.*, parsimonious) peptides were considered for quantification.

### Batch Correction and Data Preprocessing for the Brain Proteome

A total of 10,426 high-confidence master proteins were identified across all 26 TMT batches, but only proteins quantified in >50% of samples were included in subsequent analyses (n = 7787 proteins). Log2 abundances were normalized as a ratio divided by the central tendency of pooled standards (GIS). As previously applied, the batch correction was performed using a Tunable Approach for Median Polish of Ratio (https://github.com/edammer/TAMPOR), an iterative median polish algorithm for removing technical variance across batch (12). Multidimensional scaling plots were used to visualize batch contributions to variation before and after batch correction. Network connectivity was used to remove outliers, that is samples that were greater than three standard deviations away from the mean as described (12). Finally, nonparametric bootstrap regression was performed to remove the potentially confounding covariates of age, sex, and PMI. Each trait was subtracted times the median coefficient from 1000 iterations of fitting for each protein while protecting for diagnosis (Control, AsymAD, and AD).

### Consensus Weighted Gene Correlation Network Analysis

We used the cWGCNA (version 1.69) algorithm to generate a central network of co-expression modules from both brain regions (30, 31). The WGCNA::blockwiseConsensusModules function was run with soft threshold power at 7.0, deepsplit of 4, minimum module size of 30, merge cut height at 0.07, mean topological overlap matrix (TOM) denominator, using bicor correlation, signed network type, pamStage and pamRespectsDendro parameters both set to TRUE and a reassignment threshold of 0.05. This function calculates pair-wise biweight mid-correlations (bicor) between protein pairs. The resulting correlation matrix is then transformed into a signed adjacency matrix which is used to calculate a TOM, representing expression similarity across samples for all proteins in the network. This approach uses hierarchical clustering analysis as 1 minus TOM and dynamic tree cutting lends to module identification. Following construction, module eigenprotein (ME) values were defined—representative abundance values for a module that also explain modular protein covariance. Pearson correlation between proteins and MEs was used as a module membership measure, defined as kME.

### Network Preservation

We used the WGCNA::modulePreservation() function to assess the network module preservation of our current consensus network with a recent large-scale TMT network from Brodmann area 9 (BA9) (12). Zsummary composite preservation scores were obtained using the consensus network as the test network and the previous BA9 TMT network as the reference network., with 500 permutations. A random seed was set to 1 for reproducibility, and the quickCor option was set to 0.

### Gene Ontology (GO) and Cell Type Marker Enrichment Analyses for the Brain Proteome

To characterize differentially expressed proteins and co-expressed proteins based on Gene Ontology (GO) annotation we used GO Elite (version 1.2.5) as previously described (12, 25, 32), with pruned output visualization using an in-house R script. Cell type enrichment was also investigated as previously published (12, 25, 32). An in-house marker list combined previously published cell type marker lists from Sharma *et al.* (33) and Zhang *et al.* (34) were used for the cell type marker enrichment analysis for each of the five cell types assessed (neuron, astrocyte, microglia, oligodendrocyte and endothelial). If, after the lists from Sharma *et al.* and Zhang *et al.* were merged, gene symbol was assigned to two cell types, we defaulted to the cell type defined by the Zhang *et al.* list such that each gene symbol was affiliated with only one cell type. The gene symbols in the list were processed through MyGene to ensure updated nomenclature and then converted human symbols using homology lookup. Fisher’s exact tests were performed using the human cell type marker lists to determine cell type enrichment and were corrected by the Benjamini-Hochberg procedure.

### PWAS Results Module Enrichment analysis

Proteins (n = 8356) tested in the PWAS study by Yu *et al.* (14) for correlation with cognitive resilience (or decline, when negatively correlated) were split into lists of unique gene symbols representing protein gene products positively correlated (n = 645) and negatively correlated (n = 575) to cognitive resilience, and then these lists with corresponding *p* values were separately checked for enrichment in consensus TMT network modules using a permutation-based test (10,000 permutations) implemented in R with exact *p* values for the permutation tests calculated using the permp function of the statmod package. Module-specific mean *p* values for risk enrichment were determined as a *Z* score, specifically as the difference in mean *p* value of gene product proteins hitting a module at the level of gene symbol minus the mean *V*alue of genes hit in the 10,000 random replacement permutations, divided by the standard deviation of *p* value means also determined in the random permutations.

### ThT Aggregation Assay

The effect of NRN1 on A*β* 1 to A*β* 1 42 aggregation was measured by *in vitro* ThT fluorescence assay essentially as previously described (22). Recombinant human Aβ_42_ (20 μg/ml equivalent to 5 μM) from rPeptide (# A-1170-1) was incubated in 1× Tris-buffered Saline (TBS; 150 mM NaCl, 50 mM Tris-HCl, pH 7.6), and 20 μM ThT in the presence or absence of purified recombinant NRN1 (5 μg/ml or 263 nM; Abcam, ab69755) protein. The assay was conducted in 100 μl reaction volumes in quadruplicates using chilled 96-well black clear bottom plates (Corning, #3904). Fluorescence was captured at 420 Ex, 480 Em for 20 h at 15 min intervals at 37 °C using Synergy H1 (Biotek) microplate reader. ThT alone was measured and subtracted as background fluorescence. Fluorescence intensities were graphed using GraphPad prism.

### SDS-PAGE and Immunoblot Analyses

For human brain homogenates, 10 μg of protein from each sample was mixed with Laemmli sample buffer (Bio-rad) and β-mercaptoethanol, boiled at ∼95 °C for 10 min, spun briefly to collect the volume, loaded into Bolt 4 to 12% Bis-Tris gels (Invitrogen), and electrophoresed at 160 V for ∼30 min. Gels were then stained with Coomassie Blue for protein banding visualization.

For products of the ThT aggregation assay, Aβ_42_ fibrils were precipitated by centrifugation at 10,000*g*. The pellet was resuspended in 50 μl 8 M urea buffer (8 M urea, 100 mm NaHPO4, pH 8.5) and boiled in Laemmli sample buffer (BioRad, 161-0737) at 98 °C for 5 min. Proteins were resolved on Bolt 4 to 12% Bis-Tris gels (Thermo Fisher Scientific, NW04120BOX) followed by transfer to nitrocellulose membrane using iBlot 2 dry blotting system (ThermoFisher Scientific, IB21001). Membranes were incubated with StartingBlock buffer (ThermoFisher, 37543) for 30 min followed by overnight incubation at 4° in primary antibodies, Aβ (Novus, NBP11-97929) and NRN1 (Abcam, ab64186). Membranes were washed with 1× Tris-buffered saline containing 0.1% Tween 20 (TBS-T) and incubated with fluorophore-conjugated secondary antibodies (AlexaFluor-680 or AlexaFluor-800) for 1 h at room temperature. Membranes were subsequently washed three times with TBS-Tween, and images were captured using an Odyssey Infrared Imaging System (LI-COR Biosciences).

### Silver Staining

Aβ_42_ fibrils prepared in the ThT assay earlier were precipitated by centrifugation at 10,000*g*. The pellet was resuspended in 50 μl 8 M urea buffer (8 M urea, 100 mm NaHPO4, pH 8.5), and 10 μl of fibrils were boiled in Laemmli sample buffer (BioRad, 161-0737) at 98 °C for 5 min. Fibrils were run on Bolt 4 to 12% Bis-Tris gels (Thermo Fisher Scientific, NW04120BOX) and stained using a silver staining kit (Pierce, 24612) following the manufacturer’s protocols. Briefly, it was rinsed twice in ultrapure water for 5 min followed by fixation in 30% ethanol and 10% acetic acid in water. The gel was washed in 10% ethanol and water. The gel was then incubated in silver stain and developer solutions. Staining was quenched using 5% acetic acid and images were captured using a scanner.

### Primary Rat Hippocampal Culture

Primary rat hippocampal cultures were generated from E18 Sprague-Dawley rat embryos as previously described (35, 36). All experimental procedures were performed under a protocol approved by the Institutional Animal Care and Use Committee (IACUC) at the University of Alabama at Birmingham (UAB). Rats were euthanized with procedures that are consistent with the recommendations of the American Veterinary Medical Association Guidelines for the Euthanasia of Animals and approved by the UAB IACUC. Briefly, cell culture plates were coated overnight with 1 mg/ml poly-L-lysine (Sigma-Aldrich, catalog no. P2636-100MG) and rinsed with diH20. Neurons were cultured at a density of 4 × 10^5^ cells per 18-mm glass coverslip in 12-well culture plates (Fisher Scientific, catalog no. 353043). Neurons were cultured in Neurobasal medium (Fisher Scientific, catalog no. 21103-049) supplemented with B27 (Fisher Scientific, catalog no. 17504-044), conditioned by separate cultures of primary rat astrocytes and glia, in a humidified CO2 (5%) incubator at 37 °C. Neurons were treated at DIV 4 with 5 μM cytosine β-D-arabinofuranoside hydrochloride (Sigma-Aldrich, catalog no. C6645) to eliminate the presence of native astrocytes and glia on the glass coverslips. The medium was changed every 3 to 4 days with a new glia-conditioned Neurobasal medium for proper culture maintenance. At DIV 12, neurons were transfected with Lifeact-GFP plasmid to visualize the actin cytoskeleton, using Lipofectamine 2000 (Invitrogen, catalog no. 11-668-019) according to the manufacturer’s instructions. We and others have extensively used Lifeact-GFP to analyze dendritic spine density and morphology in cultured neurons and animal models under numerous experimental conditions and analysis approaches with highly consistent results (35, 36, 37, 38, 39, 40). The primary papers on Lifeact, published in Nature Methods, elegantly show that cells of all kinds exhibit normal actin dynamics when expressing Lifeact-GFP (41, 42). At DIV 14, primary hippocampal neurons were dosed with either DMSO, 500 nM Aβ_42_, 150 ng/ml recombinant neuritin (NRN1), or a combination of 500 nM Aβ_42_ plus 150 ng/ml NRN1 for 6 h. Six hours was chosen based on past studies demonstrating that Aβ_42_-induced spine loss in cultured neurons plateaus at approximately 6 h post exposure (35, 43). The concentration of Aβ_42_ oligomers was chosen based on original findings by Lacor *et al.* (43, 44) that indicated treatment of cultured rodent neurons with 500 nM synthetic Aβ_42_ oligomers allowed for synaptic uptake and neuronal interaction with Aβ_42_ oligomers. Moreover, Lacor *et al.* (43, 44) as well as our own studies demonstrate that a concentration of 500 nM Aβ_42_ induces highly reproducible dendritic spine degeneration without causing cell death. Past studies by Lacor *et al.* and our lab demonstrated that Aβ_42_ induced spine loss in primary hippocampal neurons plateaus at approximately 6 h after exposure (35, 43). Based on this, the experiments conducted herein utilized a 6-h time point. Our goal was to keep the time point consistent across all cultured neuron experiments in order to provide a collective snapshot of the neurobiology putatively ongoing within that time frame. Previous studies demonstrated that NRN1 exists predominantly in a soluble form *in vivo* (45); however, the physiological concentrations of NRN1 in the brain are unknown. The concentration of NRN1 (150 ng/ml) was chosen based on past reports indicating that exogenous application of 150 ng/ml soluble NRN1 protein (highly similar to the reagent used in this study) induced alterations in dendritic structure and physiology in cultured hippocampal neurons in the presence of Aβ (46, 47).

### Static Widefield Microscopy

On DIV 14, neurons were fixed with room temperature 2% paraformaldehyde in 0.1 M PBS, washed two times with 1× PBS, and coverslips were mounted on microscope slides (Fisher Scientific, catalog no. 12-550-15) using Vectashield mounting media (Vector Labs, catalog no. H1000). A blinded experimenter performed all microscopy. Images were captured on a Nikon Eclipse Ni upright microscope, using a Nikon Intensilight and Photometrics Coolsnap HQ2 camera to image Lifeact-GFP. Previous studies demonstrated that Lifeact-expressing neurons display normal, physiological actin dynamics, and dendritic spine morphology (41, 42). Images were captured with Nikon Elements 4.20.02 image capture software using 60X oil-immersion objective (Nikon Plan Apo, N.A. 1.40). Z-series images were acquired at 0.10 μm increments through the entire visible dendrite. Dendrites were selected for imaging by using the following criteria: (1) minimum of 25 μm from the soma; (2) no overlap with other branches; and (3) must be a secondary dendritic branch. Prior to analysis, capture images were deconvolved using Huygens Deconvolution System (16.05, Scientific Volume Imaging) with the following settings: CMLE; maximum iterations: 50; signal to noise ratio: 40; quality: 0.1. Deconvolved images were saved in.tif formation.

### Dendritic Spine Morphometry Analysis

Image analysis was performed with Neurolucida 360 (2.70.1, MBD Biosciences) based on previously described methods (35). Dendritic spine reconstruction was performed automatically using a voxel-clustering algorithm and the following parameters: outer range: 10.0 μm; minimum height: 0.5 μm; detector sensitivity 100%; minimum count: 8 voxels. Next, the experimenter manually verified that the classifier correctly identified all protrusions. When necessary, the experimenter added any protrusions semi-automatically by increasing detector sensitivity. Each dendritic protrusion was automatically classified as a dendritic filopodium, thin spine, stubby spine, or mushroom spine based on previously described morphological measurements (48). Reconstructions were collected in Neurolucida Explorer (2.70.1, MBF Biosciences) for branched structure analysis and then exported to Microsoft Excel. Spine density was calculated as the number of spines per 10 μm of dendrite length.

### Multi-Electrode Array Recording and Analysis

Single neuron electrophysiological activity was recorded using a Maestro Edge multiwell microelectrode array and Impedance system (Axion Biosystems). Before 24 h of multielectrode array (MEA) culturing, each well of a 6-well plate (Axion Biosystems, catalog no. M384-tMEA-6W-5) was coated with 1 mg/ml Poly-L-lysine (Sigma, catalog no. P2636-100MG). The next day, wells were washed with diH_2_O. E18 rat primary hippocampal neurons were harvested as described above and plated in a 6-well MEA at a density of 4 × 10^5^ cells per well. Each MEA well contained 64 extracellular recording electrodes. Neurons were cultured DIV 0 to DIV 4 in Neurocult Neuronal Plating Medium (Stemcell Technologies, catalog no. 05713) with SM1 neuronal supplement (Stemcell Technologies, catalog no. 05711). At DIV 4, the media was changed to BrainPhys Neuronal Medium (Stemcell Technologies, catalog no. 05790) with SM1 neuronal supplement. At DIV 14, a 5-min MEA prerecording was performed followed by the application of DMSO, 500 nM Aβ_42_, 150 ng/ml NRN1, or 150 ng/ml NRN1 and 500 nM Aβ_42_. After 6 h, a follow-up 5-min MEA recording was performed to determine the effects on neuronal firing. All recordings were performed while connected to a temperature-controlled heater plate (37 °C) with 5% CO_2_. All data were filtered using 0.1-Hz (high pass) and 5-kHz (low pass) Butterworth filters. Action potential thresholds were set manually for each electrode (typically >6 standard deviations from the mean signal). Sorting of distinct waveforms corresponding to multiple units on one electrode channel was completed in Offline Sorter (v. 4.0, Plexon). Further analysis of the firing rate was performed in NeuroExplorer (v. 5.0, Plexon). The mean firing frequency was calculated as spikes/second and log_10_ transformed.

### Cortical Rat Neuronal Culture, Lysis, and Proteolytic Digestion

Primary rat cortical neurons were generated from E18 Sprague-Dawley rat embryos with minor modifications (35, 36). Neurons were cultured at a density of 4 × 10^5^ cells per well in 12-well culture plates (Fisher Scientific, catalog no. 353043). Neurons were cultured in Neurobasal medium (Fisher Scientific, catalog no. 21103-049) supplemented with B27 (Fisher Scientific, catalog no. 17504-044). Culture maintenance included a half-media change every 2 to 3 days. At DIV 14, neurons were either treated with 150 ng/ml recombinant NRN1 protein (Abcam, ab69755) or vehicle-treated with diH_2_O for 6 h. NRN1 concentration was chosen based on published data that identified a plateau in exogenous NRN1-induced effects on transient potassium currents at 150 ng/ml (49). After 6 h neurons were washed 2× with 1 ml 1× phosphate-buffered saline (PBS). To harvest cells, 1 ml 1× PBS + protease inhibitor (Fisher Scientific, catalog no. 78426) was added, and the cells were centrifuged for 2300 rpm for 5 min at 4 °C. Cell pellets were lysed in 200 μl 8 M urea buffer and HALT protease and phosphatase inhibitor cocktail (1× final concentration). Lysates were sonicated with a probe sonicator three times for 10 s with 10 s intervals at 30% amplitude and cleared of cellular debris by centrifugation in a tabletop centrifuge at 18,000 rcf for 3 min at 4 °C. Protein concentration was determined by BCA assay and one-dimensional SDS-PAGE gels were run followed by Coomassie blue staining as quality control for protein integrity and equal loading before proceeding to protein digestion. Protein homogenates (50 μg) were diluted with 50 mM NH4HCO3 to a final concentration of less than 2 M urea and then treated with 1 mM DTT at 25 °C for 30 min, followed by 5 mM iodoacetamide at 25 °C for 30 min in the dark. Protein was digested with 1:100 (w/w) lysyl endopeptidase (Wako) at 25 °C for 2 h and further digested overnight with 1:50 (w/w) trypsin (Pierce) at 25 °C. The resulting peptides were desalted with a Sep-Pak C18 column (Waters) and dried under vacuum.

### TMT Labeling for the Rat Neuronal Proteome

Peptides from each individual cell line in the study and a global pooled reference internal standard (GIS) were labeled using the TMTpro 16-plex kit (ThermoFisher Cat#A44520 Lot#VH311511). Labeling was performed essentially as previously described (18, 24). Briefly, each sample (containing 100 μg of peptides) was resuspended in 100 mM TEAB buffer (100 μl). The TMT labeling reagents were equilibrated to room temperature, and anhydrous ACN (256 μl) was added to each reagent channel. Each channel was gently vortexed for 5 min, and then 41 μl from each TMT channel was transferred to the peptide solutions and allowed to incubate for 1 h at room temperature. The reaction was quenched with 5% (vol/vol) hydroxylamine (8 μl) (Pierce). All 16 channels were then combined and dried by SpeedVac (LabConco) to approximately 150 μl and diluted with 1 ml of 0.1% (vol/vol) TFA, then acidified to a final concentration of 1% (vol/vol) FA and 0.1% (vol/vol) TFA. Peptides were desalted with a 200 mg C18 Sep-Pak column (Waters). Each Sep-Pak column was activated with 3 ml of methanol, washed with 3 ml of 50% (vol/vol) ACN, and equilibrated with 2 × 3 ml of 0.1% TFA. The samples were then loaded and washed with 2 × 3 ml of 0.1% (vol/vol) TFA and 2 ml of 1% (vol/vol) FA. Elution was performed with two volumes of 1.5 ml 50% (vol/vol) ACN. The eluates were then dried to completeness. High pH fractionation was performed next as described for human samples.

### LC-MS/MS for the Rat Neuronal Proteome

All samples were analyzed with a Dionex Ultimate 3000 RSLCnano in capillary flow mode. The analytical column was a 300 μm × 150 mm ID Waters CSH with 1.7 μm beads. Mass spectrometry was performed with a high-field asymmetric waveform ion mobility spectrometry (FAIMS) Pro equipped Orbitrap Eclipse (Thermo) in positive ion mode using data-dependent acquisition with 1.5 s top speed cycles for each FAIMS compensation voltage (CV). Each cycle consisted of one full MS scan followed by as many MS/MS events that could fit within the given 1.5 s cycle time limit. MS scans were collected at a resolution of 120,000 (410–1600 m/z range, 4 × 10^5^ AGC, 50 ms maximum ion injection time, FAIMS CV of −45 and −65). All HCD MS/MS spectra were acquired at a resolution of 30,000 (0.7 m/z isolation width, 35% collision energy, 1.25 × 10^5^ AGC target, 54 ms maximum ion time, TurboTMT on). Dynamic exclusion was set to exclude previously sequenced peaks for 20 s within a 10-ppm isolation window.

### Data Search and Protein Quantification for the Rat Neuronal Proteome

All raw files (n = 96) were analyzed using the Proteome Discoverer Suite (version 2.4) Thermo Scientific). MS/MS spectra were searched against the UniProtKB rat proteome database (downloaded April 2015 with 29,370 total sequences). The Sequest HT search engine was used with the following parameters: fully tryptic specificity; maximum of two missed cleavages; minimum peptide length of 6; fixed modifications for TMT tags on lysine residues and peptide N-termini (+304.207 Da) and carbamidomethylation of cysteine residues (+57.02146 Da); variable modifications for oxidation of methionine residues (+15.99492 Da), deamidation of asparagine and glutamine (+0.984 Da), and phosphorylation of serine, threonine, and tyrosine (+79.966); and precursor mass tolerance of 10 ppm; and fragment mass tolerance of 0.05 Da. The Percolator node was used to filter PSMs to an FDR of <1%. Following spectral assignment, peptides were assembled into proteins and were further filtered based on the combined probabilities of their constituent peptides to a final FDR of 1%. A Multi-consensus was performed to group proteins identified across the individual batches. In cases of redundancy, shared peptides were assigned to the protein sequence in adherence with the principles of parsimony. A total of 125,869 peptides mapped to 9799 protein groups. Reporter ions were quantified from MS2 scans using an integration tolerance of 20 ppm with the most confident centroid setting. Only unique and razor (*i.e.*, parsimonious) peptides were considered for quantification. TMT channels 129C, 130N, and 130C correspond to NRN1-treated samples and channels 132C, 133N, 133C, and 134N correspond to vehicle-treated samples which were used for the presented results.

### Rat Neuronal Proteome Overlap With Human Consensus Modules

Human consensus module (39 modules) protein members were converted to rat symbols using the biomaRt package, and the overlap of rat neuronal proteins was determined for each module. A one-tailed Fisher exact test looking for significant overrepresentation or overlap was employed, and *p* values were corrected for multiple testing using the Benjamini–Hochberg method. R functions fisher.test() and p.adjust() were used to obtain the above statistics.

### Additional Statistical Analyses

All proteomic statistical analyses were performed in R (version 4.0.3). Box plots represent the median and 25th and 75th percentile extremes; thus the hinges of a box represent the interquartile range of the two middle quartiles of data within a group. Error bars extents are defined by the farthest data points up to 1.5 times the interquartile range away from the box hinges. Correlations were performed using the biweight midcorrelation function from the WGCNA package. Group comparisons in human brain samples were performed with one-way ANOVA with Holm post hoc correction of all comparisons. Differential expression between NRN1 and vehicle-treated neurons was determined by Student’s *t* test and corrected for multiple hypothesis testing by the permutation-based ROTS package (v1.18.0) (20) for FDR correction. The ROTS() function was run with parameters B = 100, K = 900, and seed set to 1. Differential expressions, displayed as volcano plots, were generated using the ggplot2 package. Go annotation for rat neuron proteins was performed as described for human samples. *p* values were adjusted for multiple comparisons by FDR correction where indicated.

All analyses from dendritic spine morphometric and MEA results were conducted with Prism 9.0 (GraphPad Software). Data are presented as mean ± SEM, and all graph error bars represent SEM. All statistical tests were two-tailed with threshold for statistical significance set at 0.05. Statistical comparisons on spine densities and morphologies are one-way ANOVA with Tukey’s comparison test. Statistical comparisons on mean firing rate are unpaired Student’s *t* test.

## Results

### Proteomic Measurements Align With Neuropathological Scores

**Fig. 1:**
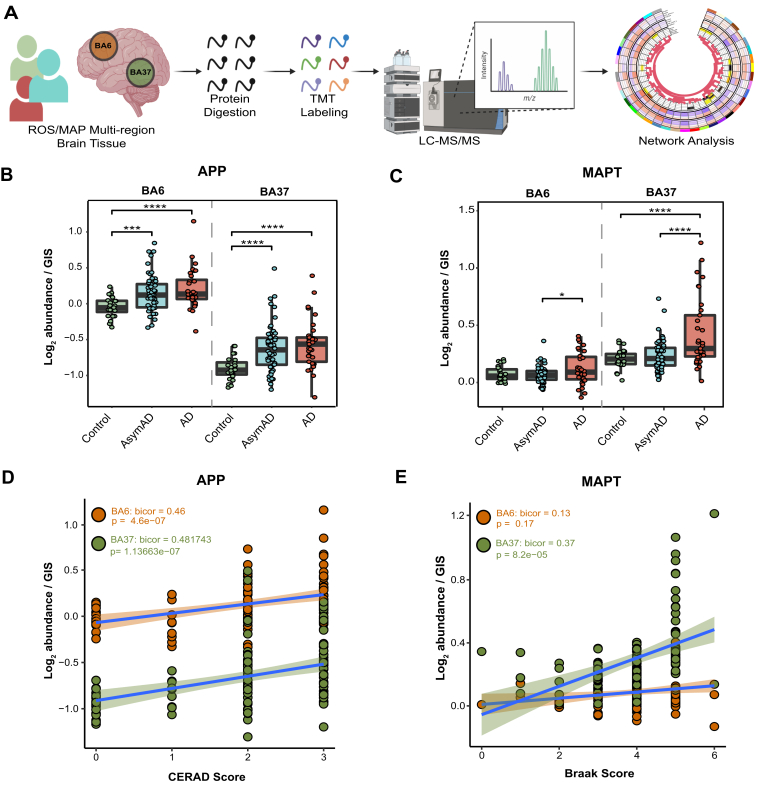
**Proteomic measurements of amyloid and tau align with region-specific neuropathological burden.***A*, schematic representation of the experimental workflow for matched human brain tissue samples across regions BA6 and BA37 from 109 ROSMAP cases that were enzymatically digested with trypsin into peptides and individually labeled with isobaric tandem mass tags (TMT) followed by LC-MS/MS. Log_2_ abundances were normalized as a ratio divided by the central tendency of pooled standards (global internal standards, GIS) and median centered. Protein abundances were analyzed using differential and co-expression methods. *B*, TMT-MS quantified APP normalized abundance is significantly increased in AsymAD and AD cases compared to Control. One-way ANOVA (BA6: F = 7.987, *p* > 0.001; BA37: F = 9.469, *p* > 0.001) with Tukey’s multiple comparisons test. *C*, TMT-MS quantified MAPT normalized abundance is significantly increased in AD. One-way ANOVA (BA6: F = 3.522, *p* < 0.05; BA37: F = 12.69, *p* > 0.001) with Tukey’s multiple comparisons test. *D*, APP normalized abundance and CERAD scores positively correlate in each brain region. Biweight midcorrelation (Bicor) and *p* value (BA6: bicor = 0.46, *p* = 4.6e-07; BA37: bicor = 0.482, *p* = 1.1e-07). Best-fit line for each region is determined by a linear model, and confidence interval is shaded around line. *E*, MAPT normalized abundance and Braak scores positively correlate in BA37. Bicor and *p* value (BA6: bicor = 0.13, *p* = 0.17; BA37: bicor = 0.37, *p* = 8.2e-05). Best-fit line for each region is determined by a linear model, and confidence interval is shaded around line. ∗*p* < 0.05, ∗∗∗*p* < 0.005, ∗∗∗∗*p* < 0.001; F, F value; Bicor, biweight midcorrelation. AD, Alzeimer’s disease.

Matched post-mortem brain tissue samples from BA6 and BA37 from 109 ROSMAP (n = 218 samples total) cases were analyzed using multiplex TMT-MS (Fig. 1*A*). BA6 is a frontal cortex area containing the premotor and supplementary motor cortices, important for roles in motor, language, and memory functions (50). BA37 resides in the temporal cortex and contains the fusiform gyrus which has been linked to disrupted language and memory function in AD (51). Cases were classified as Control, AsymAD, or AD based on semi-quantitative measures of amyloid (CERAD) and tau (Braak) deposition as well as cognitive function near the time of death (supplemental Fig. S1) (12, 18). This classification strategy, similar in concept to the A/T/N framework, which stratifies cases based on the presence or absence of Amyloid, Tau, and Neurodegeneration, allows distinction of cases with an increased neuropathological burden but intact cognitive function (52). TMT-MS–quantified protein levels were filtered for missing values in <50% of samples and adjusted for batch effects, outlier removal, and confounding effects of covariates (age, sex, and post-mortem interval or PMI) for a final expression dataset of 7787 proteins (supplemental Fig. S2).

Protein levels related to the primary AD pathologies, amyloid precursor protein (APP), and microtubule-associated protein tau (MAPT) were compared across disease groups and brain regions (Fig. 1, *B* and *C*). The APP levels served as a surrogate measurement for Aβ and as expected were significantly higher in AsymAD and AD cases compared to Controls in both brain regions (Fig. 1*B*; (12, 25)). Consistently, we also assessed individual tryptic peptides mapping to the Aβ sequence in APP. As seen at the protein level with APP, these Aβ peptides were significantly increased in both AsymAD and AD cases compared to control and positively correlated with CERAD scores in each brain region (supplemental Fig. S3). MAPT levels were significantly higher in AD compared to AsymAD in BA6 and significantly increased in AD compared to both Control and AsymAD in BA37 (Fig. 1*C*). APP levels positively correlated with CERAD scores, irrespective of the brain region, and exhibited higher baseline levels in BA6 (Fig. 1*D*). MAPT levels in BA37, but not BA6, positively correlated with Braak scores (Fig. 1*E*). These measurements align with well-established region-specific neuropathological burden observed in AD in which amyloid pathology manifests initially in neocortical regions while NFT pathology originates and intensifies in *trans*-entorhinal cortex regions before spreading to temporal and frontal cortical areas (15,16). CERAD and Braak scores are both semi-quantitative measurements that profile global plaque or NFT pathology, respectively. APP and MAPT protein abundance discussed here is quantified in a single brain region (BA6 or BA37), offering an explanation for the lack of correlation of MAPT and Braak in BA6. Collectively, these strong positive correlations of APP levels with CERAD scores and MAPT levels with Braak scores highlight the accuracy of the proteomic measurements in quantifying relevant AD neuropathological burden.

Consistent with these targeted pathology-linked proteins (APP and MAPT), the differential expression of all quantified proteins was assessed (supplemental Tables S1 and S2). As expected, proportional numbers of significantly different proteins were identified in disease groups across brain regions compared to controls (BA6: Control *versus* AsymAD = 222, Control *versus* AD = 1102; BA37: Control *versus* AsymAD = 129, Control *versus* AD = 1550). This suggests that the differences in the proteome correspond proportionately with differences in neuropathology as previously described (25). In addition, the greater number of differentially expressed proteins in BA37 compared to BA6 underscores the regional difference across the two brain regions. Our analysis provides a comprehensive list of differentially expressed proteins that align with brain region-specific pathology in AD.

### Regional Brain Co-expression Network Analysis Reveals Modules Associated With AD Pathology and Cognition

**Fig. 2:**
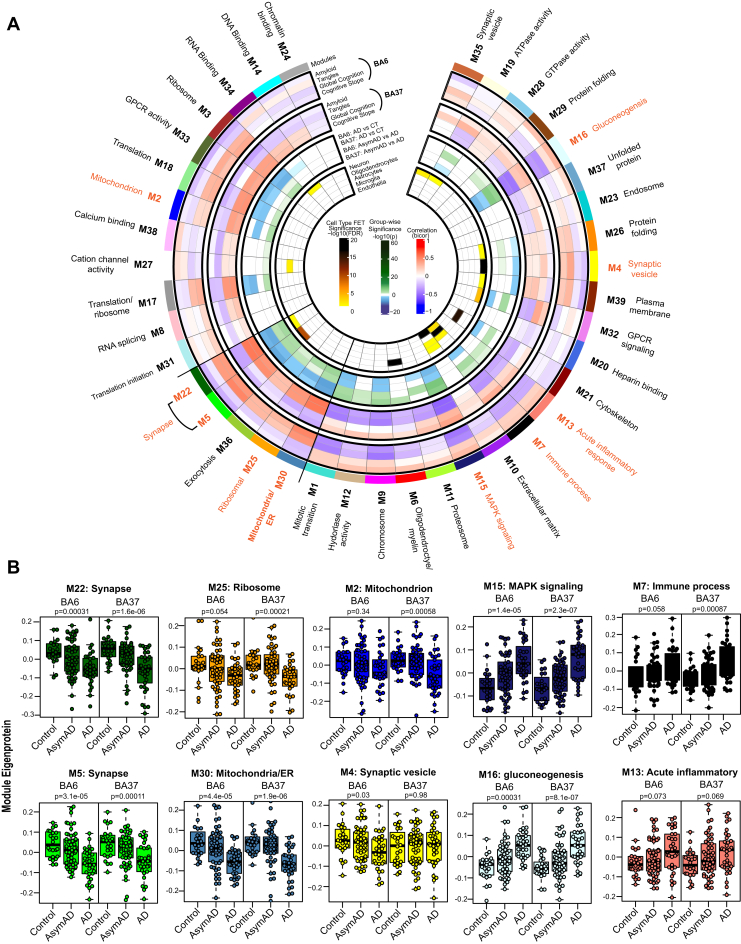
**Consensus correlation network of a multi-region human brain proteome.***A*, a consensus correlation network (cWGCNA) was constructed with 7787 proteins across BA6 and BA37 and yielded 39 co-expression protein modules. In the inner most heatmap, enrichment of cell-type markers (as determined by one-way Fisher’s exact test) for each module is visualized for neuronal, oligodendrocyte, astrocyte, microglial, and endothelial cell types. The panel outside of the cell type results highlights group-wise differences in module eigenproteins for AD *versus* Control and AsymAD *versus* AD in each brain region. The two outer most heatmaps depict the correlation (Bicor) of module eigenproteins with pathological (Amyloid and Tangle burden) and clinical (Global cognitive function and cognitive slope) phenotypes for both brain regions. Modules are identified by color and number, accompanied by top gene ontology (GO) terms representative of modular biology. Scale bars for cell-type enrichment (*darker color* indicates stronger enrichment), weighted group-wise eigenprotein difference (*darker green* correspond to a stronger positive relationship and *deeper blue* indicates a stronger negative relationship), and bidirectional module—trait relationships (*red* indicating positive correlation and *blue* indicating negative correlation) are at the center of the plot. *B*, module eigenproteins (MEs) grouped by diagnosis (Control, AsymAD, and AD) were plotted as box and whisker plots for modules of interest, chosen based on their preservation in AsymAD compared to AD and relationships to cognitive measures. MEs were compared in each brain region using one-way ANOVA, unadjusted *p* values are shown. Box plots represent median, 25th, and 75th percentiles. *Box hinges* represent the interquartile range of the two middle quartiles with a group. Error bars are based on data points 1.5 times the interquartile range from the *box hinge*. AD, Alzheimer’s disease; AysmAD, asymptomatic AD; cWGCNA, consensus weighted gene correlation network analysis; BA6, Brodmann area 6; BA37, Brodmann area 37.

A correlation network was constructed using the cWGCNA algorithm, a systems biology approach to identify biologically meaningful, co-expression patterns (30, 31). The consensus configuration allows the identification of highly preserved modules, or clusters of interconnected proteins, shared across BA6 and BA37 while retaining region-specific relationships (supplemental Fig. S2, *B* and *C*). A total of 39 co-expression modules (M1-M39) were defined, ranging in size from 36 members (M39) as the smallest and 473 members (M1) as the largest (Fig. 2*A* and supplemental Tables S3 and S4). Similar patterns of intermodule relationships were observed in BA6 and BA37 (supplemental Fig. S4). GO analysis was performed on the protein members of each consensus module, and top-ranking GO terms across multiple ontology types were considered in determining representative module biology (supplemental Table S5). To detect modules related to neuropathological burden and cognitive changes, ME (the first principle component of the expression matrix; MEs) were correlated with Aβ plaque (“Amyloid”) and NFT (“Tangles”) burden in the brain at autopsy as well as global cognitive scores and cognitive slope for each person prior to death (supplemental Tables S6 and S7). Immunohistochemistry and systematic sampling of eight brain regions were averaged to determine Amyloid and Tangle load (53). Global cognition is a composite score of 19 cognitive performance tests, and the cognitive slope is calculated based on changes in cognitive performance over time (17). To understand group-wise differences in MEs, modules were further characterized according to AD *versus* Control and AsymAD *versus* AD pairwise differences. Finally, the cell-type contribution of each module was assessed by determining cell-type marker enrichment for neuronal, oligodendrocyte, astrocyte, microglia, and endothelial cell types (supplemental Tables S8 and S9) (12).

Module preservation of the current consensus regional network was compared to a recent, large-scale network analysis of the human dorsolateral prefrontal cortex (Brodmann area 9, BA9) generated from ROSMAP and Banner cases (12). Preservation of modules from the BA9 network was checked in multiple brain regions, including the data of the current study. However, the current network configuration is a new analysis in which BA6 and BA37 are being considered in a single, shared network. Notably, approximately 95% (37/39) of the consensus modules were preserved with the previous TMT-MS network (supplemental Fig. S5), and all 39 consensus modules from this study had significant protein overlap with at least one module from the BA9 network (supplemental Fig. S5). Thus, modules generated by cWGCNA are robust and highly preserved across different cohorts and brain regions.

From the present network, we observed the interplay of module biology with cognition, disease status, and brain region. Specifically, a cluster of five modules was identified as positively correlated with cognition and were increased in AsymAD compared to AD similarly in both brain regions: M22 Synapse, M5 Synapse, M36 Exocytosis, M25 Ribosome, and M30 Mitochondria/ER (Fig. 2). We also observed modules following this pattern in only one brain region: M2 Mitochondrion was only significant in BA37 and M4 Synaptic vesicle was only significant in BA6. In contrast, M15 MAPK signaling and M16 Gluconeogenesis were the most strongly negatively correlated with cognition and decreased in AsymAD compared with AD. These findings are consistent with previous proteomic findings in BA9 where sugar metabolism and MAPK signaling modules were significantly related to cognition (12). Overall, we generated a consensus network, highly consistent with previous brain proteome network modules, that sufficiently outlined key differences and an interrelationship among clinical traits, disease groups, and even regional brain differences.

### Nomination of Resilience-Associated Modules and NRN1 as Top Protein Candidate

**Fig. 3:**
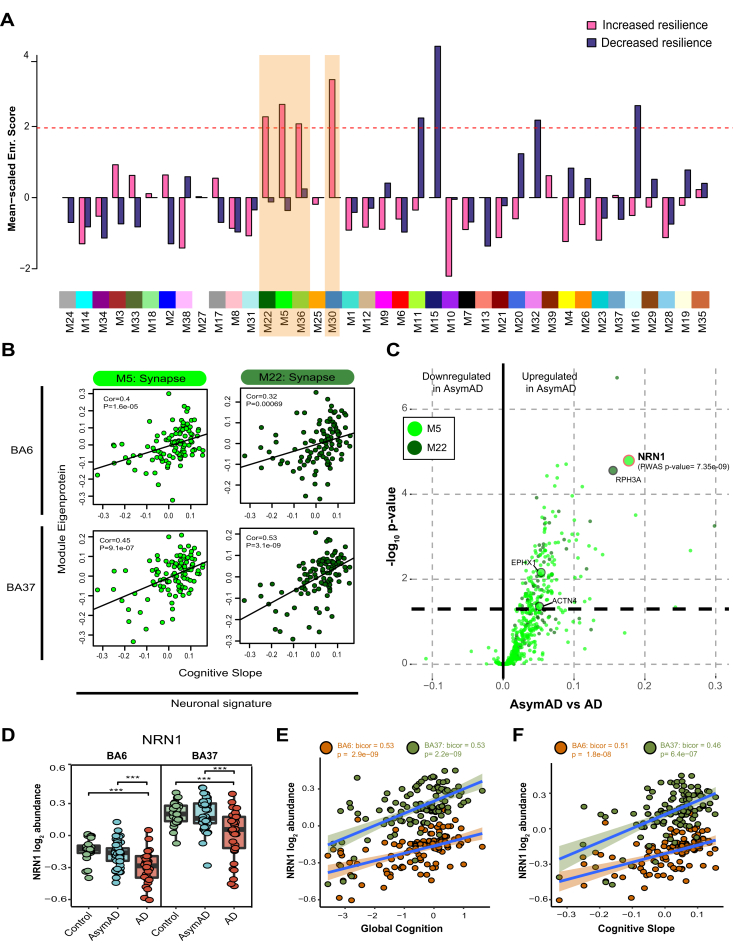
**Integrated proteomics of human brain reveals NRN1 as a top resilience candidate.***A*, significant enrichment of modules associated with increased cognitive resilience was identified by PWAS in consensus modules. The *dashed red line* illustrates the significance cutoff corresponding to a Z score of 1.96 or *p* = 0.05. Significant, increased resilience modules are highlighted in orange. *B*, PWAS significant, synaptic modules M5 and M22 positively correlate with cognitive slope, irrespective of the brain region. Bicor and *p* values (BA6: M22 cor = 0.32, M22 *p* = 0.00069, M5 cor = 0.4, M5 *p* = 1.6e-05; BA37: M22 cor = 0.53, M22 *p* = 3.1e-09, M5 cor = 0.45, M5 *p* = 9.1e-07). *C*, differential expression comparing AsymAD and AD groups from M5 and M22 module members. Protein fold-change is the x-coordinate and the −log_10_*p* value from one-way ANOVA is the y coordinate for each protein. Proteins above the *dashed line* (*p* = 0.05) are considered significantly differentially expressed. *Large circles* highlight proteins that were significant by PWAS (α = 5e-06). *D*, NRN1 abundance is significantly reduced in AD. One-way ANOVA (BA6: F = 13.25, *p* < 0.001; BA37: F = 13.68, *p* < 0.001) with Tukey test. *E*, NRN1 abundance correlates positively with global cognitive performance. Bicor and *p* values (BA6: bicor = 0.53, *p* = 2.9e-09; BA37: bicor = 0.53, *p* = 2.2e-09). *F*, NRN1 abundance correlates positively with cognitive slope. Bicor and *p* values (BA6: bicor = 0.51, *p* = 1.8e-08; BA37: bicor = 0.46, *p* = 6.4e-07). AD, Alzheimer’s disease; AysmAD, asymptomatic AD; BA6, Brodmann area 6; BA37, Brodmann area 37; NRN1, neuritin; PWAS, proteome-wide association study.

To increase external validity while expanding upon previous findings and unbiasedly nominating consensus modules linked to resilience, we integrated the results from a recent brain PWAS of cognition that evaluated the association of cortical protein abundances with cognitive resilience from an independent TMT-MS proteomic analysis of ROSMAP tissues adjusted for AD pathologies (14). Samples from the current study and previous PWAS were processed and analyzed separately, with only 30 cases shared across cohorts. Higher abundances of proteins related to slower rates of cognitive decline were considered to confer greater resilience while a higher abundance of proteins associated with a faster rate of cognitive decline was considered to confer less resilience. Four modules were identified as significantly enriched with proteins conferring greater cognitive resilience: M22 Synapse, M5 Synapse, M36 Exocytosis, and M30 Mitochondria/ER (Fig. 3A and supplemental Table S10). In addition, four modules were found to be significantly enriched for proteins conferring less cognitive resilience: M11 Proteosome, M15 MAPK signaling, M32 GPCR signaling, and M16 Gluconeogenesis (Supplemental Table S11). Of the modules associated with greater resilience, the protein constituents of M5 and M22 were strongly representative of synaptic biology and enriched for neuronal markers. To further confirm the association of M5 and M22 to cognitive preservation, MEs were correlated with cognitive slope and indicated a strong positive correlation in both brain regions (Fig. 3*B*). Consistently, differential expression comparing AsymAD with AD of proteins specific to M5 and M22 exhibited a strong bias toward an increase or upregulation of proteins in AsymAD (Fig. 3*C*). Two proteins significantly upregulated in AsymAD were also significant in the aforementioned PWAS of cognition (14), NRN1 and Rabphilin-3A (RPH3A). NRN1 was more significantly differentially expressed than RPH3A and NRN1 was the most significant protein associated with increased cognitive resilience in the PWAS. NRN1 abundance in both brain regions was compared across disease groups and indicated comparable levels in AsymAD and controls but NRN1 was significantly downregulated in AD (Fig. 3*D*). NRN1 abundance also strongly, positively correlated with cognitive measures including global cognition and cognitive slope (Global cognition: BA6 *p* value = 2.9e-09, BA37 *p* value = 2.2e-09; Cognitive slope: BA6 *p* value = 1.8e-08, BA37 *p* value = 6.4e-07 Fig. 3, *E* and *F*). Furthermore, variance partition analysis of global cognition in BA6 and BA37 identified NRN1 as the top (B6: ∼26% variance explained) and second (BA37: ∼38% variance explained) protein explaining the highest variance in global cognition (supplemental Fig. S6; supplemental Tables S12 and S13).

In summary, the integration of independent human proteomic datasets prioritized protein modules associated with cognitive resilience across two brain regions. Four modules (M22, M5, M36, and M30) were significantly enriched for proteins linked to greater cognitive resilience in life. Two modules (M22 and M5) captured biology related to synaptic integrity and were found to be vulnerable in AD but preserved in asymptomatic cases. NRN1 was identified as a hub protein in M5 and NRN1 abundance levels were significantly increased in AsymAD cases compared to AD. Notably, the differences between AsymAD and AD across brain regions observed in this study *via* TMT-MS is highly consistent with other large-scale label-free proteomic datasets from several patient cohorts (18). The reproducibility and rigor of this analysis strongly support our hypothesis that NRN1 is a protein mediator of cognitive resilience.

### NRN1 Prevents Aβ_42_-Induced Dendritic Spine Degeneration

**Fig. 4:**
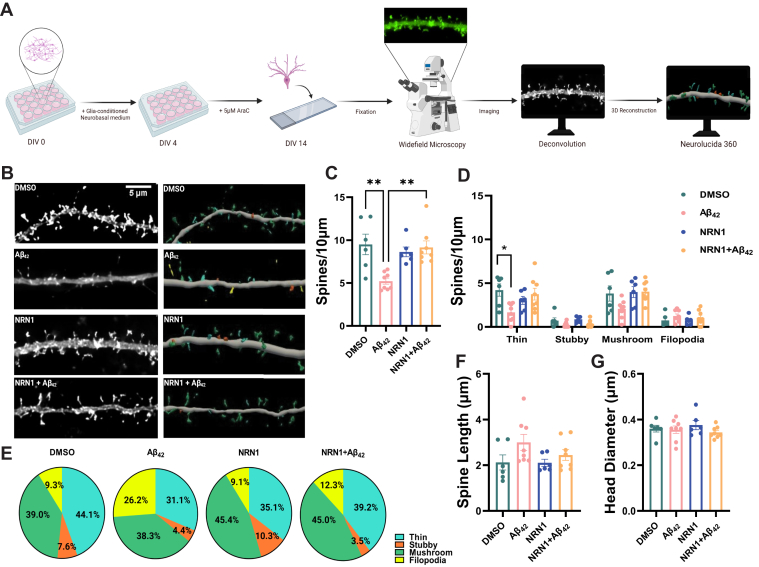
**Aβ**_**42**_**-induced dendritic spine degeneration is blocked by NRN1.***A*, schematic representation of primary rat hippocampal neuron treatment and dendritic spine morphometric analysis. *B*, representative maximum-intensity wide-field fluorescent images of hippocampal neurons after deconvolution (*left*). Corresponding three-dimensional reconstructions of dendrites generated in Neurolucida 360 (*right*), with dendritic spines color-coded by spine type (*blue* = thin, *orange* = stubby, *green* = mushroom, *yellow* = filopodia). Scale bar, 5 μm. N = 6 to 8 neurons (one dendrite per neuron) were analyzed per experimental condition. *C*, dendritic spine density in hippocampal neurons exposed to DMSO, 500 nM Aβ_42_, 150 ng/ml NRN1, or 150 ng/ml NRN1 and 500 nM Aβ_42_. ∗∗*p* < 0.01 (DMSO *versus* Aβ_42_, actual *p* = 0.0025) (Aβ_42_*versus* NRN1 + Aβ_42_, actual *p* = 0.0026) by one-way ANOVA with Tukey’s test. ∗*p* < 0.05 (Aβ_42_*versus* NRN1, actual *p* = 0.0177) by one-way ANOVA with Tukey’s test. *D*, dendritic spine density of thin, stubby, or mushroom spines per 10 μm. ∗*p* < 0.05 (DMSO *versus* Aβ42, actual *p* = 0.0218) by one-way ANOVA with Tukey’s test. (Thin, Aβ_42_*versus* NRN1+Aβ_42_, actual *p* = 0.0501) (Mushroom, DMSO *versus* Aβ_42_, actual *p* = 0.1514) (Mushroom, Aβ_42_*versus* NRN1+Aβ_42_, actual *p* = 0.0598) by one-way ANOVA with Tukey’s test. *E*, dendritic spine type frequency in hippocampal neurons exposed to DMSO, 500 nM Aβ_42_, 150 ng/ml NRN1, or 150 ng/ml NRN1 and 500 nM Aβ_42_. *F*, overall dendritic spine length and (*G*) head diameter. Related data are shown in supplemental Fig. S7. *Box plots* represent median, 25th, and 75th percentiles. *Box hinges* represent the interquartile range of the two middle quartiles with a group. Error bars are based on data points 1.5 times the interquartile range from the *box hinge*. Aβ, amyloid-beta; NRN1, neuritin.

The preservation of dendritic spines is hypothesized to maintain memory and information processing in resilient patients who harbor high levels of Aβ pathology but are cognitively normal (9, 54). Numerous studies indicate that Aβ can induce dendritic spine degeneration in cellular and animal models of AD (35, 43, 55, 56). Henceforth, protecting spines from Aβ represents a rational therapeutic strategy to promote resilience and delay dementia onset. Past studies provided evidence that NRN1 exists predominantly as a soluble form *in vivo* and exerts neurotrophic effects on synaptic maintenance and neuronal survival (45, 57, 58). Based on our network analysis, we hypothesized that NRN1 could protect against Aβ-induced dendritic spine degeneration. To test this, rat hippocampal neurons were isolated at E18 and cultured at high density on glass coverslips. To visualize dendritic architecture, neurons were transiently transfected with a plasmid encoding Lifeact-GFP at DIV 12. Cultures were treated with NRN1 or co-treated with NRN1 and Aβ_42_ oligomers for 6 h, then fixed, and processed for widefield microscopy followed by three-dimensional image reconstructions for dendritic spine morphometric analysis (Fig. 4*A*). Consistent with previous reports (35), spine density was reduced significantly after exposure to Aβ_42_ in comparison to DMSO controls; however, co-treatment with NRN1 prevented Aβ_42_-induced spine degeneration (Fig. 4, *B* and *C*). Examination of dendritic spine morphologic subclasses revealed that Aβ_42_ exposure significantly decreased thin spine density in comparison to DMSO controls, however, these detrimental effects were blocked in the presence of NRN1 (Fig. 4*D*). Notably, the proportion of thin spines was increased with NRN1 treatment compared to DMSO, while Aβ_42_ promoted an increase in the proportion of dendritic filopodia (Fig. 4*E*). Exposure to Aβ_42_ and/or NRN1 did not significantly alter dendritic spine length or head diameter in comparison to DMSO controls (Fig. 4, *F* and *G* and supplemental Fig. S7). These findings suggest that NRN1 can protect against Aβ_42_-induced dendritic spine loss.

To exclude the possibility that NRN1 directly binds to soluble Aβ_42_ oligomers and in turn neutralizes each protein’s independent effects in primary neurons, we performed an *in vitro* amyloid aggregation assay. Human recombinant Aβ_42_ fibrilization was measured by thioflavin T (ThT) fluorescence in the presence or absence of NRN1 for 20 consecutive hours (supplemental Fig. S8*A*). There were no distinguishable differences in self-assembly and aggregation between Aβ_42_ alone or Aβ_42_ and NRN1 together. Following the fluorometric assay, soluble and pellet fractions were probed *via* Western blot and silver stain (supplemental Fig. S8, *B* and *C*). Nearly all NRN1 immunoreactivity was detected in the soluble fraction whereas Aβ_42_ was primarily concentrated in the pellet fraction. Importantly, the molar concentration of Aβ_42_ was 10-fold greater in the aggregation assay and the molar concentration of NRN1 was >30-fold greater, suggesting that even at very high concentrations NRN1 does not impede Aβ_42_ fibrilization. These studies suggest that it is highly unlikely an artifact of NRN1 and Aβ_42_ directly binding immediately prior to exposure on primary neurons could account for NRN1’s preventative role in Aβ_42_-induced dendritic spine loss.

### NRN1 Protects Against Aβ_42_-Induced Neuronal Hyperexcitability

**Fig. 5:**
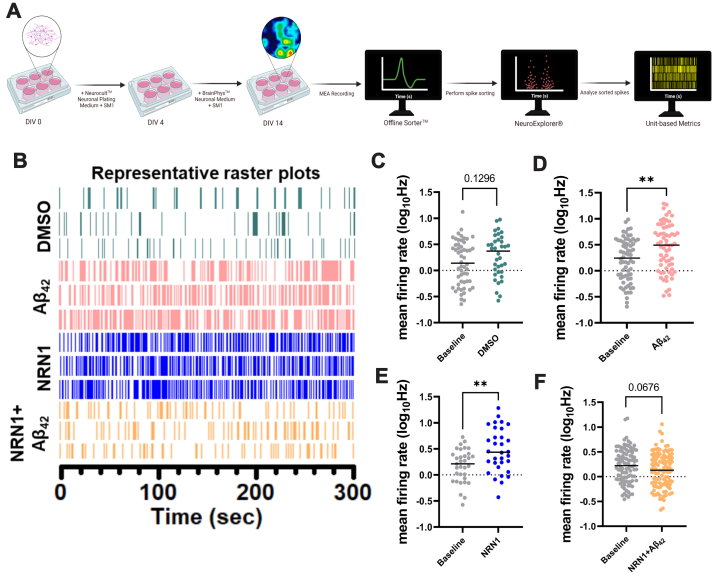
**NRN1 protects against Aβ**_**42**_**-induced neuronal hyperexcitability.***A*, schematic representation of primary rat hippocampal neuron treatment and single neuron electrophysiology analysis. *B*, representative raster plots from three units after exposure to DMSO, 500 nM Aβ_42_, 150 ng/ml NRN1, or 150 ng/ml NRN1 and 500 nM Aβ_42_. *C*, mean firing rate at DIV14 in hippocampal neurons treated with DMSO, compared to baseline (n = 36–54 neurons, unpaired Student’s *t* test; *p* = 0.1296). *D*, mean firing rate at DIV14 in hippocampal neurons treated with 500 nM Aβ_42_, compared to baseline (n = 65–68 neurons, unpaired Student’s *t* test; *p* = 0.0022). *E*, mean firing rate at DIV14 in hippocampal neurons treated with 150 ng/ml NRN1, compared to baseline (n = 32–33 neurons, unpaired Student’s *t* test; *p* = 0.0023). *F*, mean firing rate at DIV14 in hippocampal neurons treated with 150 ng/ml NRN1 and 500 nM Aβ_42_, compared to baseline (n = 100–107 neurons, unpaired Student’s *t* test; *p* = 0.0676). Aβ, amyloid-beta; NRN1, neuritin.

Aβ-induced dendritic degeneration and spine loss cause reductions in the overall area and volume of neurons, rendering them more electrically compact (35, 55). The loss of dendritic spines and the overall surface area of the neuron induces hyperexcitability, which consequently drives abnormal circuit synchronization and cognitive impairment in AD mouse models and patients (55, 59, 60). To test whether NRN1 is protective against Aβ_42_-induced neuronal hyperexcitability, we seeded rat primary hippocampal neurons on MEAs and performed baseline recordings at DIV 14. Action potential frequency, referred to as mean firing rate, was measured to assess neuronal excitability. Immediately after the baseline recording, neurons were exposed to DMSO, Aβ_42_, NRN1, and/or NRN1 plus Aβ_42_ for 6 h followed by a second recording (Fig. 5*A*). DMSO did not increase mean firing rates in comparison to the baseline (Fig. 5, *B* and *C*). Consistent with previous findings (35), Aβ_42_ significantly increased mean firing rates in comparison to baseline (Fig. 5, *B* and *D*). While NRN1 significantly increased mean firing rates in comparison to baseline, simultaneous exposure to Aβ_42_ and NRN1 was comparable to baseline (Fig. 5, *B*, *E*, and *F*). The total number of active neurons per experimental group could not account for the effects on mean firing rates (supplemental Fig. S9). These results reproduce past studies indicating that Aβ_42_-induced dendritic spine loss in cultured neurons causes hyperexcitability (35). Notably, NRN1 exposure did not alter spine density or morphology (Fig. 4); therefore, the increase in mean firing rate that was exhibited following NRN1 treatment must be the result of a different mechanism (Fig. 5*E*). Together, these findings indicate that the dendritic spine resilience provided by NRN1 is protective against Aβ_42_-induced hyperexcitability.

### NRN1 Treatment Alters the Proteome in Cultured Neurons

**Fig. 6:**
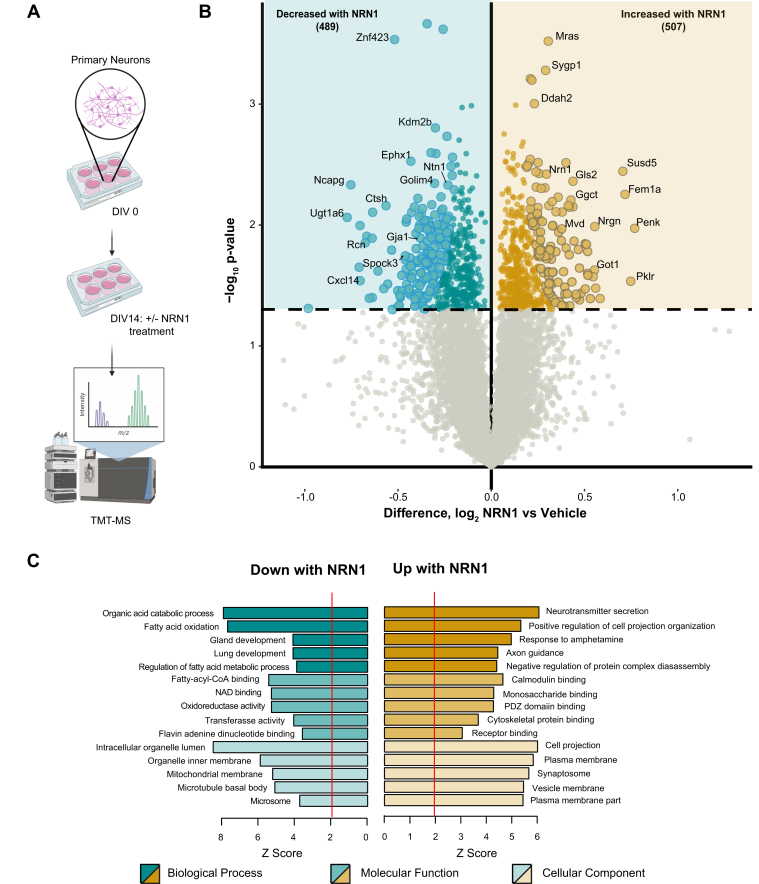
**NRN1 treatment induces changes in the neuronal proteome related to broad synaptic functions.***A*, schematic representation of rat primary cortical neuronal culture workflow in which neurons were maintained in neurobasal medium for 14 days, treated with 150 ng/ml of NRN1 and analyzed *via* TMT-MS. *B*, differential protein expression between NRN1-treated and vehicle-treated neurons (n = 8238 proteins). Proteins above the *dashed line* (*p* = 0.05) are considered significantly differentially expressed. Student’s *t* test was used to calculate *p* values. Large spots are based on reproducibility-optimized test statistic (ROTS) correction of differentially expressed proteins with 10% or less false discovery rate (FDR). *C*, gene ontology of significantly differentially expressed proteins in NRN1 treated neurons. A Z-score above 1.96 was considered significant (*p* < 0.05). NRN1, neuritin; TMT-MS, tandem mass tag mass spectrometry.

To identify proteins and broader pathways impacted by NRN1 treatment, rat primary neurons were treated with NRN1 recombinant protein at DIV14 for 6 h at the same concentration as previously tested in the dendritic spine and MEA assays (Fig. 6*A*). Following treatment, cells were lysed and prepared for TMT-MS analysis. A total of 8238 proteins were quantified and used for differential expression analysis (supplemental Table S14). Comparing NRN1-treated and vehicle-treated neurons, 507 proteins were significantly increased, and 489 proteins were significantly decreased following NRN1 treatment (Fig. 6*B* and supplemental Table S15). Of these, 216 were below a 10% FDR following correction (large spots, Fig. 6*B*). NRN1 was identified among proteins significantly increased in the NRN1 treatment group. GO analysis of significantly changed proteins found a strong bias of synaptic and cell projection functions upregulated with NRN1 exposure (Fig. 6*C*). In addition, proteins involved in functions related to oxidation and metabolic processes were decreased following NRN1 treatment. These results support previously observed functions of NRN1 in promoting synaptic function (61). Our findings provide a reference of downstream and putative co-regulated proteins that are altered by exposure to NRN1. Based on this analysis, it is possible to infer protein mediators that could be driving the increase in neuronal firing that was observed in our MEA experiments. Furthermore, the pathways decreased following NRN1 treatment were related to metabolism and cellular energetics, which are systems often dysregulated and increased in AD (12). This supports the hypothesis of NRN1 as a dual-action molecular effector that may increase proteins typically vulnerable in AD while decreasing proteins that are aberrantly increased in AD.

### NRN1 Engages Protein Targets Linked to Cognitive Resilience in Human Brain

**Fig. 7:**
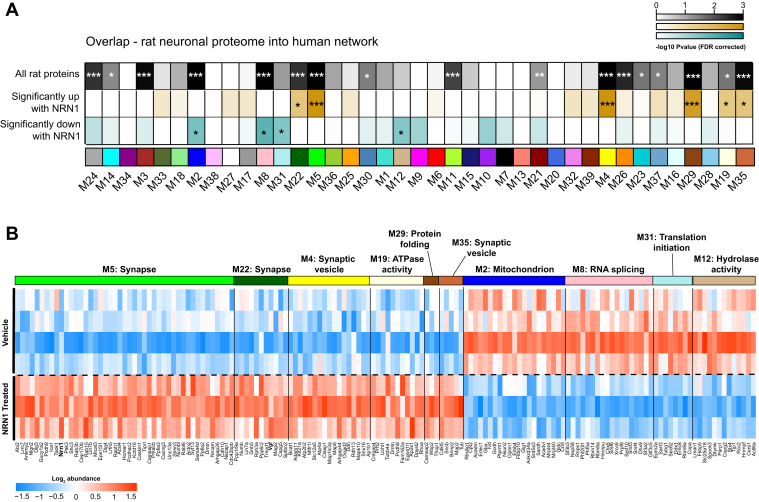
**NRN1 engages proteins within modules linked to cognitive resilience in human brain.***A*, to directly compare NRN1-induced changes in the context of human biology, a Fisher’s exact test was used to calculate significant enrichment of proteins from the entire rat proteome (*top row*), significantly increased with NRN1 (*middle row*) and significantly decreased with NRN1 (*bottom row*) treatment across the 39 human consensus modules (0.05 > *p* > 0.01 = ∗, 0.01 > *p* > 0.005 = ∗∗, *p* < 0.005 = ∗∗∗). *B*, proteins significantly impacted by NRN1 treatment that overlap with human modules were visualized as a heatmap. Rat protein abundance was compared using Bicor across NRN1-treated and vehicle-treated groups. NRN1, neuritin.

The ability of a nominated resilience protein candidate to engage relevant human biology is critically important to the clinical translation of the target. Therefore, we applied an integrative analysis to resolve how NRN1-driven changes that were observed in the rat neuronal proteome related to changes in the human AD brain proteome. The significance of protein overlap between the rat neuronal proteome and individual modules within the human brain consensus network was determined by one-tailed Fisher’s exact test (Fig. 7*A* and supplemental Table S16). The rat neuronal proteomic data was subset into three for this analysis and included: (I) all proteins quantified, (II) only those that were significantly increased following NRN1 treatment, and (III) only those that were significantly decreased following NRN1 treatment. This analysis revealed 17 of the 39 modules with statistically significant overlap from rat neuronal proteins into the human brain network (Fig. 7*A*-top row, *p* < 0.05 FDR corrected). Seven human brain modules were enriched with proteins that were significantly differentially expressed in rat neurons with NRN1 treatment (middle and bottom rows). Human modules linked to synaptic biology, M22 Synapse, M5 Synapse, M4 Synaptic vesicle, and M19 ATPase activity, in the human brain network were enriched for proteins increased following NRN1 treatment. In contrast, human modules M8 RNA splicing, M31 Translation initiation, and M12 Hydrolase activity were enriched for proteins decreased following NRN1 treatment. The majority of proteins significantly upregulated by NRN1 treatment in the rat neuronal proteome overlapped with human modules M5 and M22 (Fig. 7*B*). Notably, M5 and M22 were enriched with neuronal markers and identified as top resilience-associated modules (Fig. 3, *A* and *B*). Further, nearly all proteins increased by NRN1 in the rat neuronal proteome were significantly increased in the human asymptomatic cases (supplemental Table S17) and significantly correlated with cognitive slope (58 out of 83 or ∼70%, *p* value ≤ 0.05; supplemental Table S18). These findings provide evidence to support the hypothesis that NRN1 is capable of engaging relevant human neurobiology that is related to AD.

## Discussion

In the present study, we implemented an integrative pipeline that pairs systems-level nomination in multiple human brain regions with experimental mechanistic validation in a primary cell model. Our efforts rigorously validate and extend work from our group and others in evaluating key resilience-associated proteins and pathways. This approach enables both unbiased profiling and bidirectional integration of molecular and clinical data. Following the current framework, TMT-MS–based proteomic data from two independent studies and a total of three brain regions were incorporated to characterize communities of proteins from an in-depth proteomic dataset strongly related to cognitive resilience. NRN1, a neurotrophic factor previously reported for its association with resilience and synaptic function, was identified as a hub protein in the human consensus network and functionally validated for synaptic resilience against Aβ. To define overlapping neurobiology between NRN1’s effects on primary neurons and humans, TMT-MS proteomic data from the model system was fed back into the human brain proteome to identify convergent pathways relevant to resilience.

Correlation networks have been applied successfully to many biological and translational questions and have demonstrated validity in identifying candidate biomarkers and therapeutic targets (30, 62). Herein, cWGCNA resolved 39 co-expression modules across two brain regions from Control, AsymAD, and AD cases from ROSMAP. Applying the consensus configuration of WGCNA for matched brain tissues from the same cases identified protein communities shared across both BA6 and BA37. ME correlation with pathological and clinical traits further illuminated patterns of preservation in asymptomatic cases related to synaptic biology, cellular energetics, and protein translation. Importantly, the majority of modules identified in this study preserve a recent, large-scale network analysis, generated under different parameters, which included over 1000 cases from multiple institutions, supporting the strength and reproducibility of our findings (12). Results from an independent proteome-wide association study of cognition were then integrated to outline resilience-associated modules in our network, which included four modules significantly enriched for proteins conferring increased resilience. Among these, M5 and M22 were the most significantly enriched for synaptic biology and displayed a strong positive correlation with cognitive performance in life. NRN1, a hub of M5, has been identified as a top protein candidate of resilience previously by its relationship with cognitive trajectory (14), which is corroborated in the current study by its preservation in AsymAD cases and correlation with elevated cognitive function in life. NRN1, also known as candidate plasticity gene 15 (*CPG15*), is a neurotrophic factor that was initially discovered in a screen to identify genes involved in activity-dependent synaptic plasticity in the rat dentate gyrus (63). Over the past two decades, the role of NRN1 in regulating neurodevelopment, specifically the formation of axonal arbors and dendritic branching, has been extensively studied (46, 57, 64, 65, 66, 67, 68, 69). In adult brain, NRN1 strongly correlates with synaptic maturation, long-term stability, and activity-related plasticity (61, 63, 66, 67, 68, 69). Importantly, NRN1 was identified among proteins previously shown in multiple studies to relate to increased cognitive function and resilience to AD, including VGF, NPTX2, and RPH3A (12, 13, 14). The established link between synaptic loss and cognitive impairment in AD and the predominance of synaptic proteins in our top resilience-associated modules, warrants examining the impact of NRN1 on synaptic integrity and maintenance as foundational to determining NRN1’s role in resilience.

Dendritic spines are small actin-rich protrusions off dendrites that serve as the postsynaptic sites of the majority of excitatory synapses in the brain. Spines exhibit remarkable variability in size, shape, and density along the length of dendritic branches (70, 71, 72). Spine structure is inseparably linked to spine function and spines are classified based on their three-dimensional morphology as stubby, mushroom, thin, or filopodia (73, 74). Cognitive decline associated with aging is hypothesized to be driven by subtle alterations in dendritic spine density and morphology in mammals. Thin spine loss occurs with age in the dorsolateral prefrontal cortex and correlates with worsening cognitive performance (54, 75, 76, 77). Our past studies revealed that dendritic spine density is comparable in asymptomatic AD cases and controls but dramatically reduced in AD, indicating that spine density correlates strongly with cognitive resilience (9, 54). Further, we hypothesize that changes in the abundance of synaptic proteins, however minimal, are necessary to support the maintenance of synapses and spines in a toxic environment of Aβ and tau. Synapse numbers or spine density are likely the predominant cellular component that drives resilience, and these compartments presumably require synaptic proteins, like NRN1 and others, to maintain dendritic structure and plasticity. Future studies that investigate synapse or spine loss in dynamic disease models could shed more light on these mechanisms, enhanced by the ability to genetically alter protein levels in mice.

In parallel, patients with AD can exhibit high rates of epileptic seizure activity which is associated with accelerated cognitive decline (59, 60, 78). In APP-transgenic mice, epileptiform activity is an indicator of network hyperexcitability which is driven by the degeneration of hippocampal pyramidal neurons’ dendrites and dendritic spines (55). Loss of dendrites and spines reduces the total surface area of the cell and renders the neuron more electrically compact. In a compact neuron, synapse currents are translated more frequently which leads to increased action potential output, consequently inducing neuronal hyperexcitability and aberrant circuit synchronization (79). Similar to APP transgenic mice, exogenously applied Aβ_42_ oligomers can induce dendritic spine degeneration which subsequently causes hyperexcitability in cultured rodent hippocampal neurons (35). The results herein indicate that NRN1 suppresses Aβ_42_-induced hyperexcitability by preventing Aβ_42_-induced dendritic spine degeneration. In cultures treated with NRN1 alone, alterations in spine density or morphology were not observed, therefore NRN1-mediated increased mean action potential firing rates are not due to changes in spine density or morphology. We hypothesize that the elevation in mean firing rates is due to NRN1-mediated modification of the synaptic proteome; for instance, Figure 6*C* indicates gene ontology of significantly differentially expressed proteins in NRN1-treated neurons with “neurotransmitter secretion” as a top term. While our data suggest that NRN1 prevents Aβ_42_-induced hyperexcitability by rescuing spine degeneration, it is possible that treatment with Aβ_42_ oligomers results in detrimental effects on synaptic protein signaling pathways that counterbalance the protein-based mechanisms that are responsible for NRN1-induced increases in neuronal firing rates (80, 81). Henceforth, the application of both NRN1 and Aβ_42_ results in a leveling of neuronal activity that would be otherwise increased with NRN1 or Aβ_42_ alone. Notably, Choi *et al.* (47) showed that overexpression of NRN1 in cultured hippocampal neurons increased mini excitatory postsynaptic current frequency, which mirrors our findings that NRN1 alone increased the action potential firing rate. Furthermore, electrophysiology studies by An *et al.* (46) demonstrated that brain infusion of recombinant NRN1 (similar to the reagents used in this study) into Tg2576 APP transgenic mice rescued deficits in hippocampal long-term potentiation in the Schaffer collateral pathway. Collectively, these findings support the promise of NRN1 as a therapeutic target to support synaptic mechanisms of resiliency in the preclinical stages of AD. Determining whether NRN1 can suppress neuronal injury that is induced by pathologic tau is an important future question. One general hypothesis in the field is that pathologic tau induces synapse silencing without causing overt destruction of dendritic structure. Silent synapses lack functional α-amino-3-hydroxy-5-methyl-4-isoxazolepropionic acid receptors (AMPAR) rendering the synapse inactive (82, 83). The vulnerability of AMPARs to tau pathology can drive synaptic dysfunction in age-related tauopathies (84, 85, 86). While tau accumulation in dendritic spines reduces neuronal activity and surface AMPAR, tau does not alter synaptic density *in vitro* tauopathy models. Moreover, tau has not been reported to induce spine loss in primary hippocampal neuron cultures or organotypic slices (87, 88, 89). However, a caveat to these studies is that most of the tauopathy models that examine tau’s role in synaptotoxicity utilize the expression of human tau with familial Frontotemporal Dementia mutations.

Despite the advantages of TMT for multiplex analysis, the quantification of isobaric tags at the MS2 level has been hampered by co-isolation and co-fragmentation of interfering ions, resulting in inaccurate or suppressed TMT ratios (90, 91). This co-isolation problem can be mitigated by deep off-line fractionation and/or synchronous precursor selection (SPS)-based MS3 (SPS-MS3) quantification, both of which decrease TMT reporter ion suppression effects. Although we chose to use MS2 scans for TMT quantification in this study, these samples were all offline fractionated using high pH prior to LC-MS/MS analysis, helping to minimize peptide co-isolation. In addition, we only used quantitation from peptide spectral matches with 50% or less isolation interference (supplemental Fig. S10). Of note, approximately 90% of all spectra had 50% or less interference and nearly 70% had less than 25% interference, further increasing our confidence in quantitative accuracy. Furthermore, applying cWGCNA ensures that the biological relevance we are interpreting is not due to potential quantitative inconsistencies because the changes observed are based on the cumulated levels of a community of proteins in a module rather than individual protein abundances.

The ROSMAP studies are information-rich longitudinal aging studies that have invaluably contributed to understanding the complexity of aging and disease-related changes over time. However, this cohort is primarily made up of non-Latino white participants and historically lacks equal representation from diverse populations. Recent reports indicate Black and Hispanic populations are disproportionately more likely to have AD compared to older white Americans (92), which highlights a potential limitation of the current study. In addition to population demographics, the use of multiple definitions of resilience and how researchers identify this group adds complexity to generalizable interpretation of findings (7, 8). Based on a previously published stratification measures (12, 18), the current study used a combination of pathological and cognitive metrics to differentiate asymptomatic from symptomatic cases by imposing cutoffs that would identify potentially resilient cases. However, these selection criteria may not segregate cases that are resilient from those in a preclinical phase. Further, there may be more to learn from cases not captured by this strategy. Another potential limitation of the current study is that NRN1 neuroprotection was only assessed for Aβ insult and not tau. Quantitative neuropathological studies indicate that asymptomatic cases typically have lower levels of tau pathology but comparable levels of amyloid burden in the brain at autopsy compared to symptomatic AD cases (10). Thus, understanding the impact of NRN1 on Aβ insult is highly relevant to the pathological context observed in resilient brains. Future work investigating the interaction or effects of NRN1 on tau neuropathology may provide additional insights into NRN1 neuroprotection relevant to at-risk populations.

Conventional benchtop-to-bedside strategies for identifying therapeutic targets have generated an abundance of data in clinical trial settings, but unfortunately, often fail. Reverse translation, or bedside-to-benchtop, begins with human observational studies and works backward to pinpoint potential mechanisms and therapeutic targets for investigation. This paradigm allows information from clinical and laboratory settings to follow a cyclical process instead of a linear one and thereby is tunable and more likely to lead to successful clinical interventions (93). In the current study, we use human postmortem brain proteomic data with incorporated antemortem clinical phenotypic data (*e.g.*, cognitive trajectory in life) to characterize protein modules important for resilience to AD. NRN1 was targeted in this analysis and validated for neuroprotective efficacy in a neuronal model system. Finally, findings from our experimental models were reintegrated back into our human data to generate a distinct collection of proteins and associated biology linked to cognitive resilience in humans with high confidence. Further studies are necessary to expand these findings and elaborate on the possible impacts of NRN1 in the context of the complexity of AD. Overall, this study followed an integrative, non-linear pipeline for rigorous validation and extension of resilience-associated proteins similar to the reverse translation paradigm. The current work provides a valuable framework for investigating molecular and physiological underpinnings of resilience directed from patient samples and cognitive changes in life.

## Data Availability

The results published here are in whole or in part based on data obtained from the AMP-AD Knowledge Portal (https://adknowledgeportal.synapse.org). The AMP-AD Knowledge Portal is a platform for accessing data, analyses and tools generated by the AMP-AD Target Discovery Program and other programs supported by the National Institute on Aging to enable open-science practices and accelerate translational learning. The data, analyses and tools are shared early in the research cycle without a publication embargo on secondary use. Data are available for general research use according to the following requirements for data access and data attribution (https://adknowledgeportal.synapse.org/#/DataAccess/Instructions). Additional ROSMAP resources can be requested at www.radc.rush.edu. Raw mass spectrometry data and database search results (.pdResult files) from the human frontal/temporal cortex and rat proteome were also co-deposited to the ProteomeXchange Consortium *via* the PRIDE partner repository (94) with the dataset identifier PXD040224 and PXD040867, respectively and www.synapse.org (SynID: syn23301293, syn25006620 and syn31749523). The identified and quantified protein information for the human frontal/temporal cortex prior to bath correction is provided in supplemental Table S19 and in supplemental Table S14 for the rat proteome.

## Supplemental data

This article contains supplemental data (12).

## Conflict of interest

The authors declare no competing interests.

## Supplemental Data

Supplemental Figure S1 **Case classification traits distribution.***A*, CERAD scores are significantly increased in AsymAD and AD cases compared to control. *B*, Braak scores are significantly different across all three groups. *C*, last mini mental state exam (MMSE) scores are significantly reduced in AD compared to control and AsymAD.

Supplemental Figure S2 **Percent coverage and TMT batch correction across BA6 and BA37.***A*, percent protein coverage for all quantified high confidence, master proteins (n = 10,426) and the those present in at least 50% of all cases in both brain regions (n = 7787) after outlier removal. *B* and *C*, a median polish batch correction approach was implemented to remove technical batch variance across the 26 TMT 11-plex batches. *B*, multidimensional scaling (MDS) plots visualize original log_2_ transformed protein abundances, normalized to the pooled global internal standards (GIS). *C*, MDS of batch-corrected normalized log_2_ abundance after 175 iterations. Samples are color-coded by batch. *D* and *E*, distribution of log2 abundance data before (*D*) and after (*E*) batch correction.

Supplemental Figure S3 **Amyloid beta peptide measurements.***A*, fully tryptic peptide mapping to the Aβ region (residue 6–16) is significantly increased in AsymAD and AD compared to control cases. One-way ANOVA (BA6: F = 8.671, *p* < 0.001; BA37: F = 12.23, *p* < 0.001) with Tukey multiple comparisons test. *B*, fully tryptic peptide mapping to the Aβ region (17–28) is significantly increased in AsymAD and AD compared to control and AD is significantly increased compared to AsymAD in BA37. One-way ANOVA (BA6: F = 9.963, *p* < 0.001; BA37: F = 13.03, *p* < 0.001) with Tukey multiple comparisons test. *C*, Aβ peptide (6–16) positively correlates with CERAD scores in each brain region. Biweight (bircor) and *p* value (BA6: bicor = 0.46, *p* = 5e-07; BA37: bicor = 0.44, *p* = 1.7e-06). Best fit line for each regional correlation determined by linear mode, confidence interval is shaded around line. *D*, Aβ peptide (17–28) positively correlates with CERAD scores in each brain region. Bicor and *p* value (BA6: bicor = 0.49, *p* = 1.2e-07; BA37: bicor = 0.48, *p* = 2e-07). Best fit line for each regional correlation determined by linear mode, confidence interval is shaded around line.

Supplemental Figure S4 **Consensus modules are highly preserved in BA6 and BA37.***A*, modules eigenproteins were correlated to visualize inter-module relationships in BA6 and BA37, respectively. Heat blocks along the diagonal could be observed similarly in both brain regions. *B*, mean preservation relationship for each eigenprotein was calculated for the consensus network, with mean preservation of 0.92 indicating very high preservation. *C*, preservation adjacency of the consensus network, visualized as a heatmap, further support that most relationships in the network across both brain regions are highly preserved and biologically meaningful.

Supplemental Figure S5 **Consensus network preservation.***A*, zsummary indicates nearly all consensus modules preserve with previous BA9 TMT network modules reported in Johnson *et al.* Module Zsummary greater than or equal to 1.96 (q = 0.05, *dashed blue line*) are considered preserved and modules with Zsummary of 10 or higher (q = 1e-23, *dashed red line*) are considered highly preserved. *B*, overrepresentation analysis of consensus modules members with previous BA9 TMT module members. −log_10_ FDR corrected overlap values are shown. The heatmap threshold is at a 10% FDR (0.1).

Supplemental Figure S6 **Percent variance in protein expression explained by global cognition.** A LINEAR mixed model approach was implemented to estimate the percent variance explained by proteins in relationship to diagnosis, CERAD score, Braak score and global cognition across region, respectively. The rank order in the percent variation in protein expression explained by global cognition (top 20 proteins) were plotted for BA6 (*A*) and BA37 (*B*).

Supplemental Figure S7 **Analysis of dendritic spine length and head diameter among thin, stubby, and mushroom spines, and filopodia.***A*, dendritic spine length of thin, stubby, or mushroom spines, and filopodia. *B*, dendritic spine head diameter of thin, stubby, or mushroom spines, and filopodia. Box plots represent median, 25th and 75th percentiles. *Box hinges* represent the interquartile range of the two middle quartiles with a group. Error bars are based on data points 1.5 times the interquartile range from the *box hinge*.

Supplemental Figure S8 **Aggregation of Aβ in the presence or absence of NRN1.***A*, fibrillation curves of 20 μg/ml Aβ_42_ alone and 20 μg/ml Aβ_42_ + 5 μg/ml NRN1, thioflavin T (ThT) alone was recorded and subtracted as background. Relative fluorescent units (RFU) were recorded every 15 min for 20 h. Points are quadruplicate means ± SEM. *B*, Western blot of soluble and pelleted fractions of assay products probed for Aβ_42_ and NRN1. High molecular weight (HMW) fibrils are observed at the top of the gel. *C*, silver stain of soluble and pelleted fractions of assay products.

Supplemental Figure S9 **Total number of active neurons per microelectrode array.***A*, the total number of active neurons per well at DIV 14 in hippocampal neurons treated with DMSO, compared to baseline (n = 3–4 wells with 64 electrodes/well, unpaired Student’s *t* test; *p* = 0.8339). Data are means + SEM. *B*, the total number of active neurons per well at DIV 14 in hippocampal neurons treated with 500 nM Aβ_42_, compared to baseline (n = 5 wells with 64 electrodes/well, unpaired Student’s *t* test; *p* = 0.8878). Data are means + SEM. *C*, the total number of active neurons per well at DIV 14 in hippocampal neurons treated with 150 ng/ml NRN1, compared to baseline (n = 6 wells with 64 electrodes/well, unpaired Student’s *t* test; *p* = 0.9539). Data are means + SEM. *D*, the total number of active neurons per well at DIV 14 in hippocampal neurons treated with 150 ng/ml NRN1 and 500 nM Aβ_42_, compared to baseline (n = 6 wells with 64 electrodes/well, unpaired Student’s *t* test; *p* = 0.7035). Data are means + SEM.

Supplemental Figure S10 **Isolation interference of peptide spectral matches.***A*, percent isolation interference of all peptide spectral matches identified in BA6 and BA37 brain regions. Only spectra with less than 50% interference were used for quantitation. For interference less than 50%, approximately 90% of all spectra fell within this range and approximately 68% had less than 25% interference.
