# Supplementary figures and images for: Integrated Proteomics to Understand the Role of Neuritin (NRN1) as a Mediator of Cognitive Resilience to Alzheimer’s Disease
Source: Mol Cell Proteomics. 2023 Apr 5;22(5):100542. doi: 10.1016/j.mcpro.2023.100542 (PMC10233303; doi:10.1016/j.mcpro.2023.100542)

Supplemental Figure 1

**A**

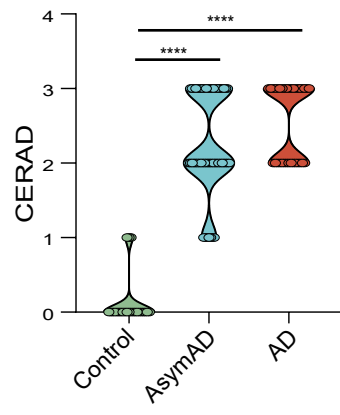

**B**

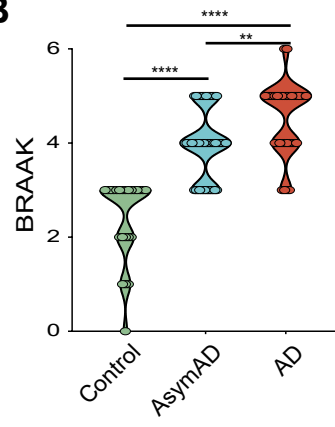

**C**

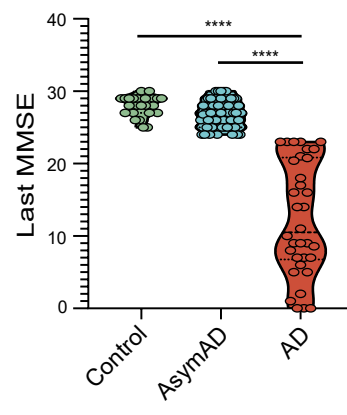

Supplement: Supplemental Figure S1 — Case classification traits distribution.A, CERAD scores are significantly increased in AsymAD and AD cases compared to control. B, Braak scores are significantly different across all three groups. C, last mini mental state exam (MMSE) scores are significantly reduced in AD compared to control and AsymAD. [file mmc1.pdf]

Supplemental Figure 2

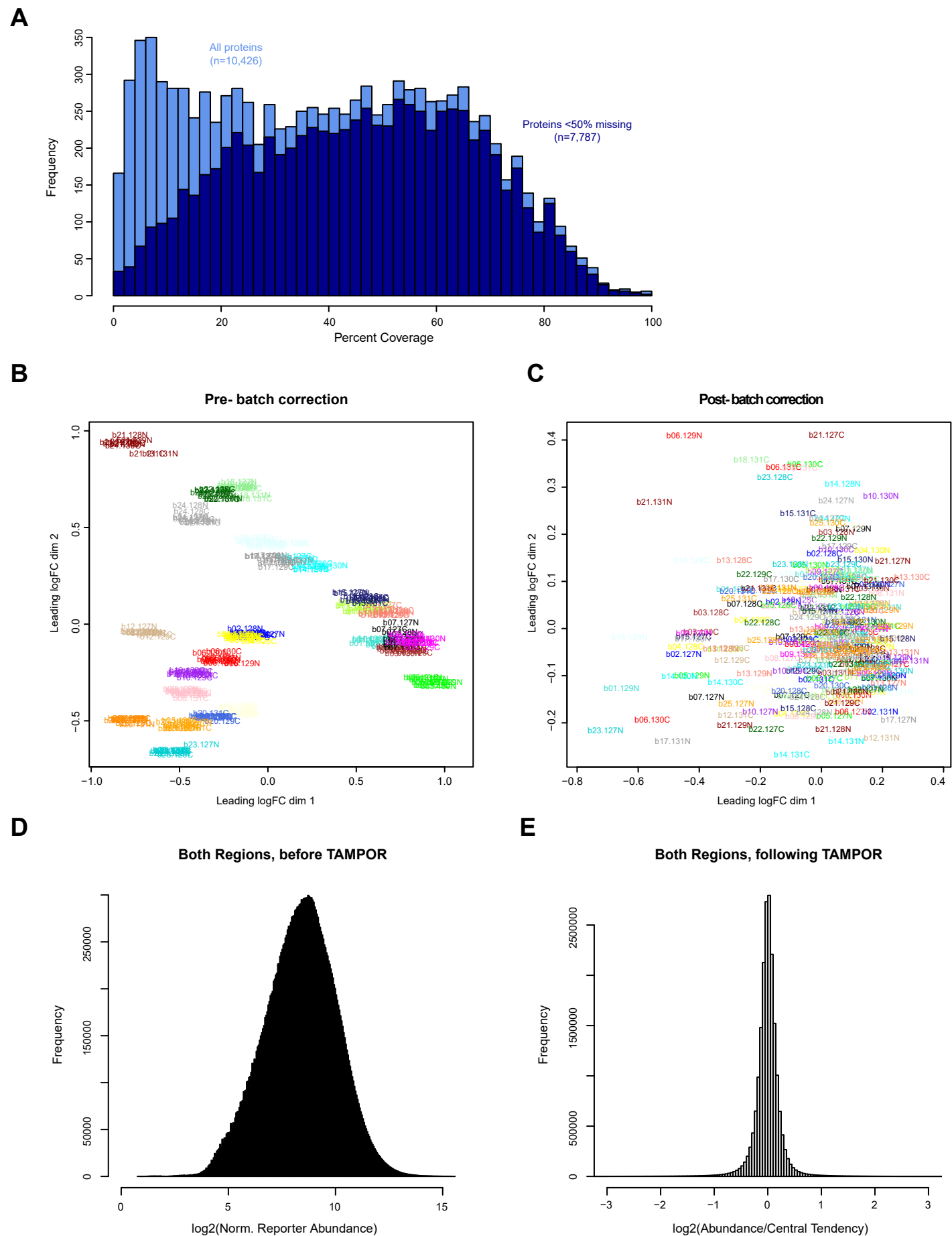

Supplement: Supplemental Figure S2 — Percent coverage and TMT batch correction across BA6 and BA37.A, percent protein coverage for all quantified high confidence, master proteins (n = 10,426) and the those present in at least 50% of all cases in both brain regions (n = 7787) after outlier removal. B and C, a median polish batch correction approach was implemented to remove technical batch variance across the 26 TMT 11-plex batches. B, multidimensional scaling (MDS) plots visualize original log2 transformed protein abundances, normalized to the pooled global internal standards (GIS). C, MDS of batch-corrected normalized log2 abundance after 175 iterations. Samples are color-coded by batch. D and E, distribution of log2 abundance data before (D) and after (E) batch correction. [file mmc2.pdf]

Supplemental Figure 3

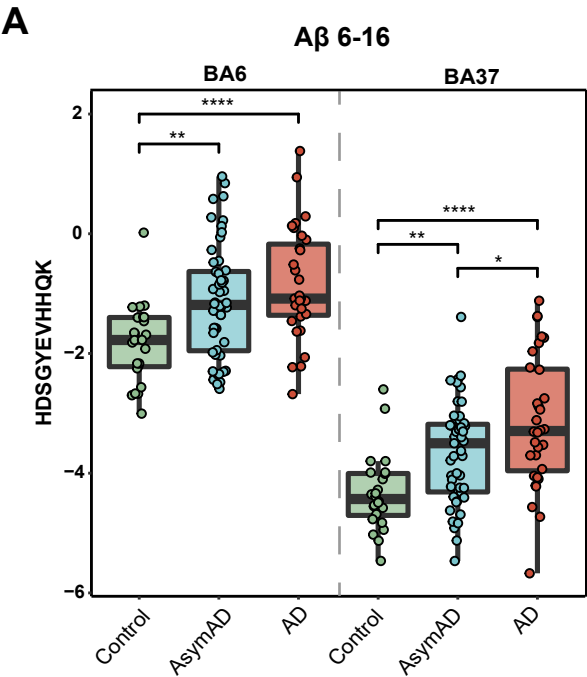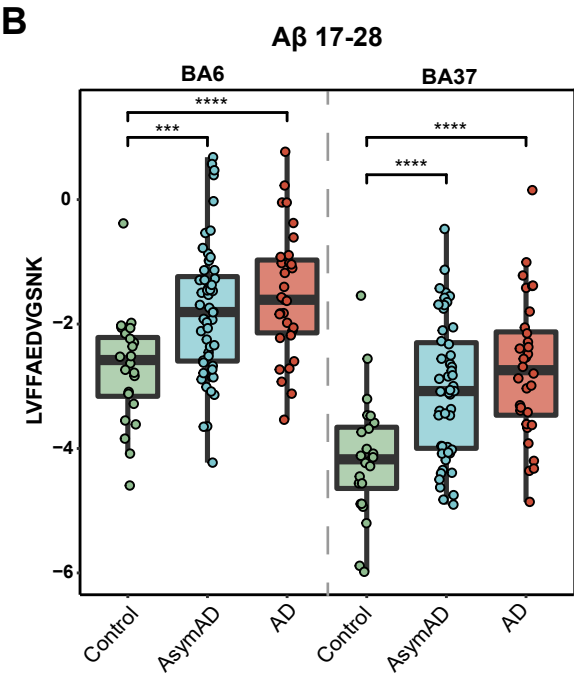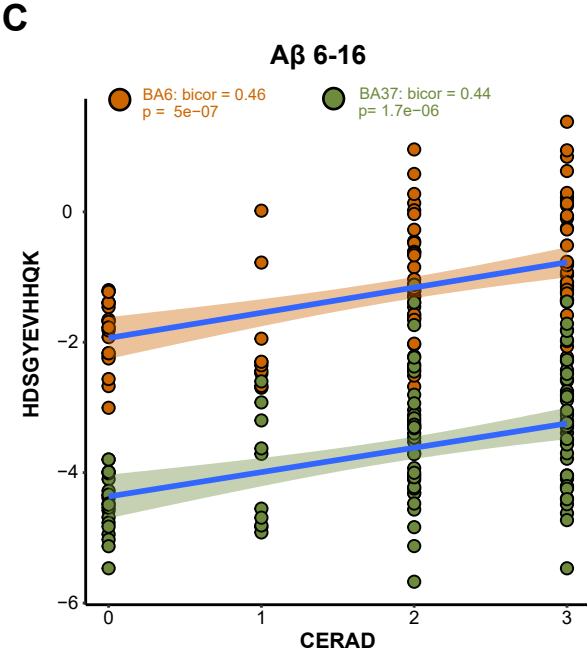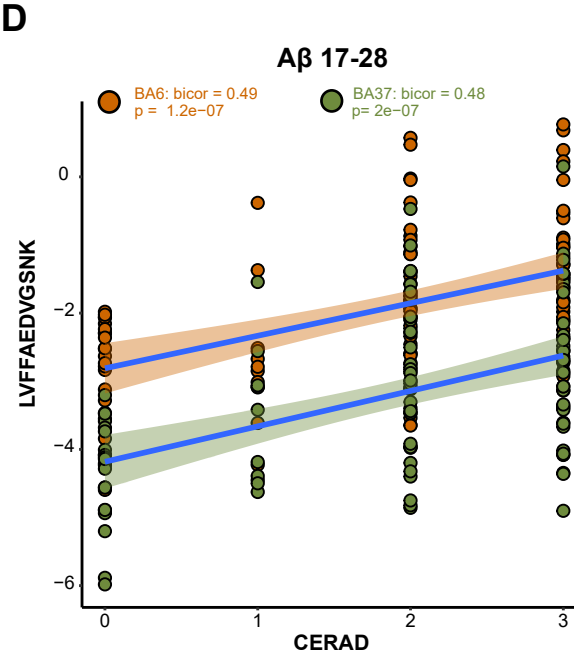

Supplement: Supplemental Figure S3 — Amyloid beta peptide measurements.A, fully tryptic peptide mapping to the Aβ region (residue 6–16) is significantly increased in AsymAD and AD compared to control cases. One-way ANOVA (BA6: F = 8.671, p < 0.001; BA37: F = 12.23, p < 0.001) with Tukey multiple comparisons test. B, fully tryptic peptide mapping to the Aβ region (17–28) is significantly increased in AsymAD and AD compared to control and AD is significantly increased compared to AsymAD in BA37. One-way ANOVA (BA6: F = 9.963, p < 0.001; BA37: F = 13.03, p < 0.001) with Tukey multiple comparisons test. C, Aβ peptide (6–16) positively correlates with CERAD scores in each brain region. Biweight (bircor) and p value (BA6: bicor = 0.46, p = 5e-07; BA37: bicor = 0.44, p = 1.7e-06). Best fit line for each regional correlation determined by linear mode, confidence interval is shaded around line. D, Aβ peptide (17–28) positively correlates with CERAD scores in each brain region. Bicor and p value (BA6: bicor = 0.49, p = 1.2e-07; BA37: bicor = 0.48, p = 2e-07). Best fit line for each regional correlation determined by linear mode, confidence interval is shaded around line. [file mmc3.pdf]

Supplemental Figure 4

A

BA6

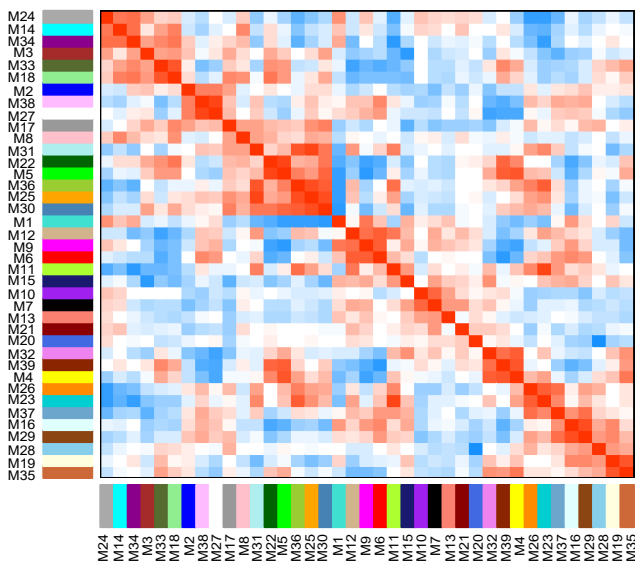

BA37

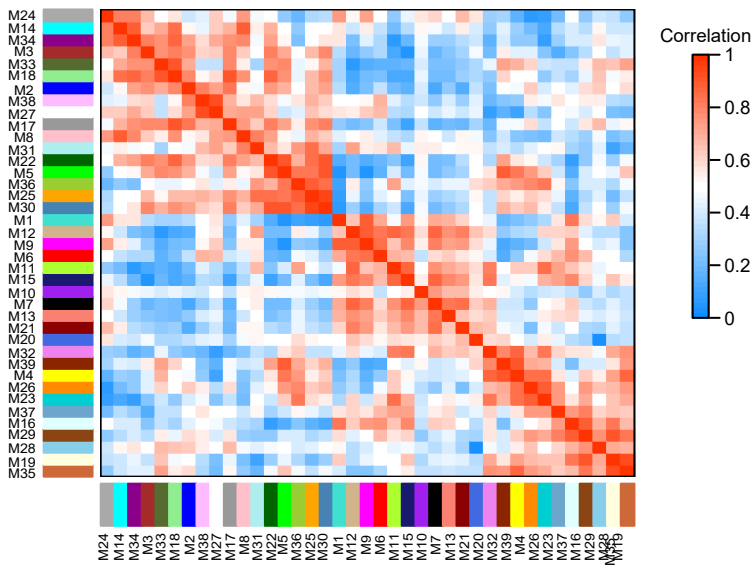

B

Mean pres.= 0.92

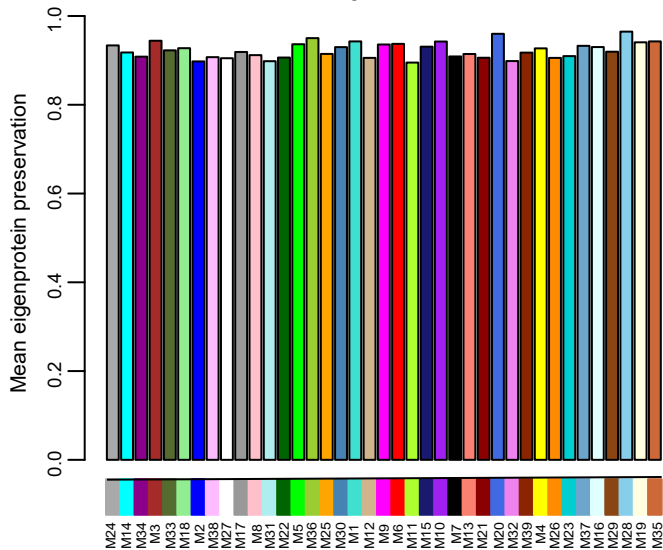

C

Preservation Adjacency

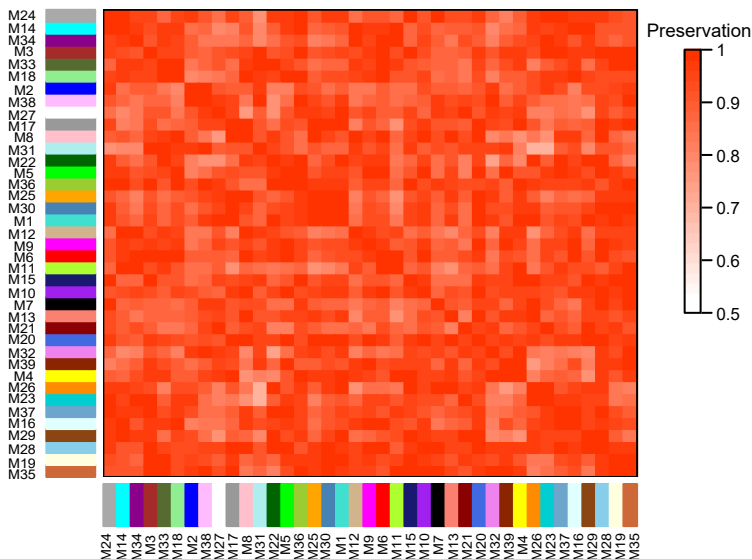

Supplement: Supplemental Figure S4 — Consensus modules are highly preserved in BA6 and BA37.A, modules eigenproteins were correlated to visualize inter-module relationships in BA6 and BA37, respectively. Heat blocks along the diagonal could be observed similarly in both brain regions. B, mean preservation relationship for each eigenprotein was calculated for the consensus network, with mean preservation of 0.92 indicating very high preservation. C, preservation adjacency of the consensus network, visualized as a heatmap, further support that most relationships in the network across both brain regions are highly preserved and biologically meaningful. [file mmc4.pdf]

Supplemental Figure 5

A

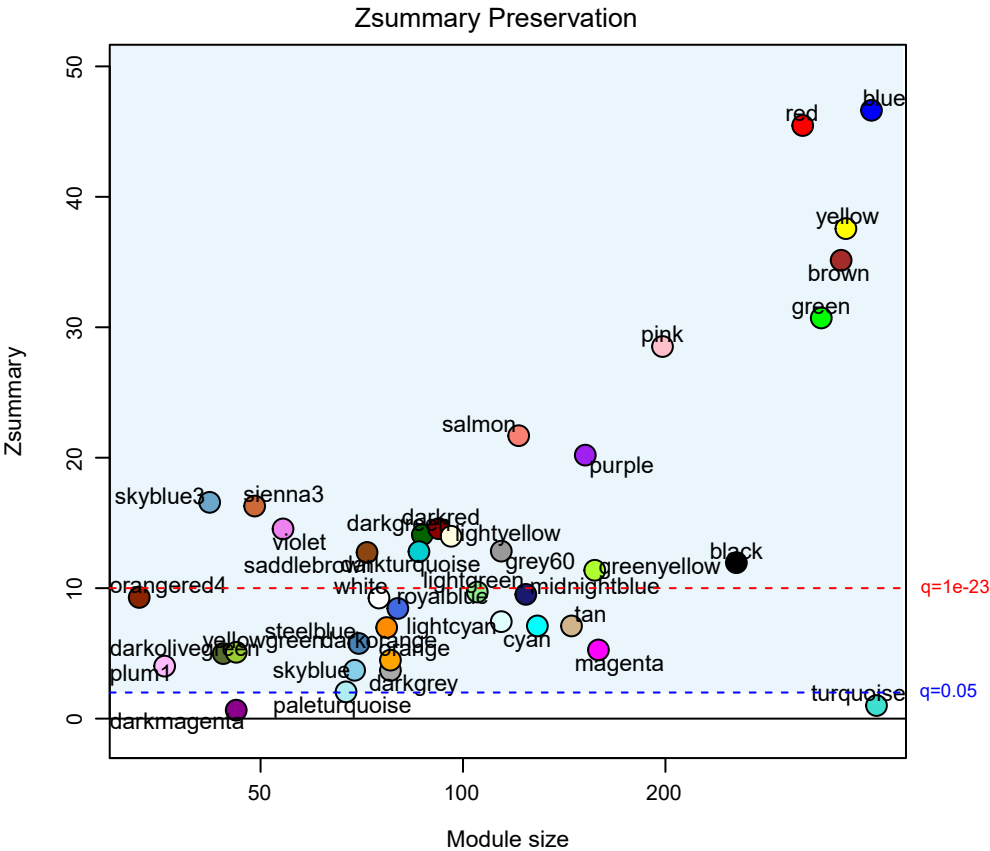

B

Consensus network module overlap with BA9 TMT network modules

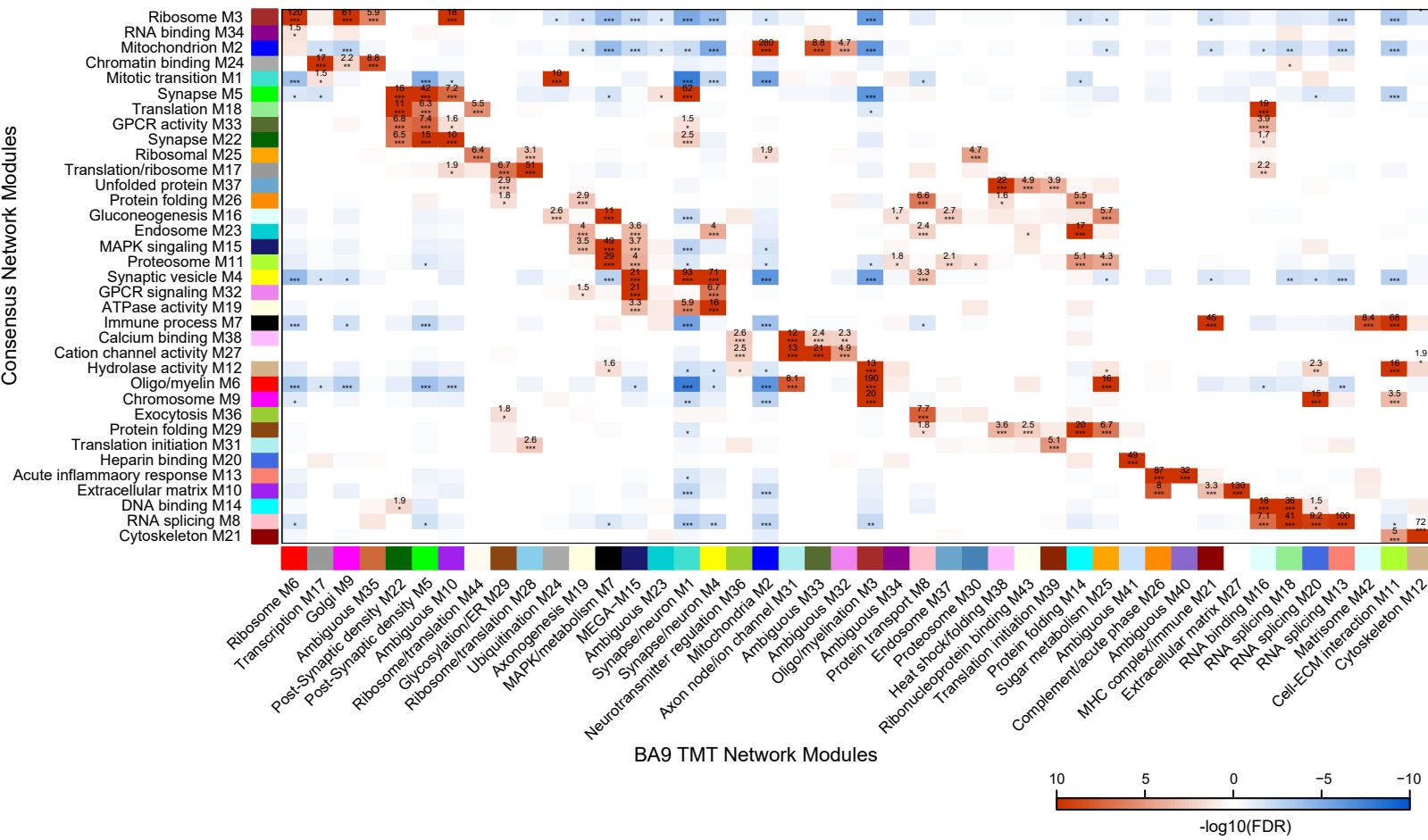

Supplement: Supplemental Figure S5 — Consensus network preservation.A, zsummary indicates nearly all consensus modules preserve with previous BA9 TMT network modules reported in Johnson et al. Module Zsummary greater than or equal to 1.96 (q = 0.05, dashed blue line) are considered preserved and modules with Zsummary of 10 or higher (q = 1e-23, dashed red line) are considered highly preserved. B, overrepresentation analysis of consensus modules members with previous BA9 TMT module members. −log10 FDR corrected overlap values are shown. The heatmap threshold is at a 10% FDR (0.1). [file mmc5.pdf]

Supplemental Figure 6

A

BA6

Global Cognition

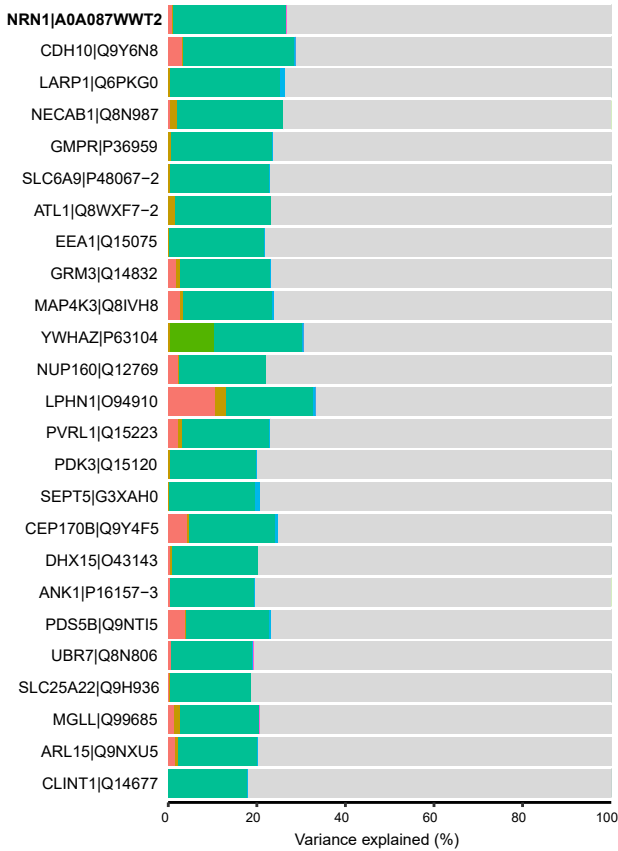

B

BA37

Global Cognition

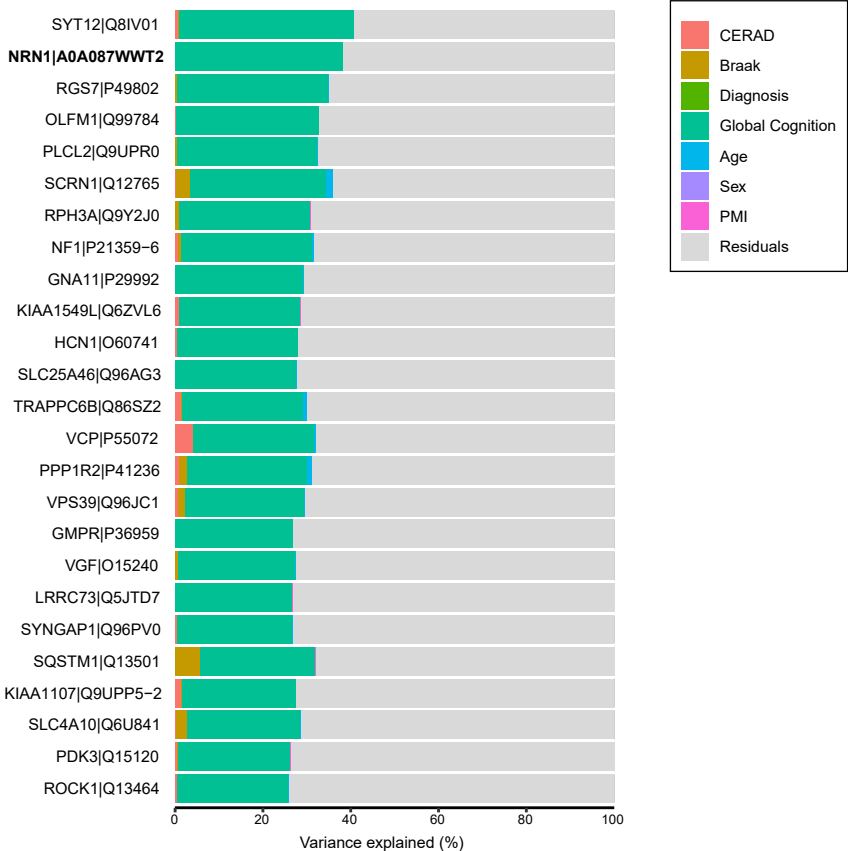

Supplement: Supplemental Figure S6 — Percent variance in protein expression explained by global cognition. A LINEAR mixed model approach was implemented to estimate the percent variance explained by proteins in relationship to diagnosis, CERAD score, Braak score and global cognition across region, respectively. The rank order in the percent variation in protein expression explained by global cognition (top 20 proteins) were plotted for BA6 (A) and BA37 (B). [file mmc6.pdf]

Supplemental Figure 7

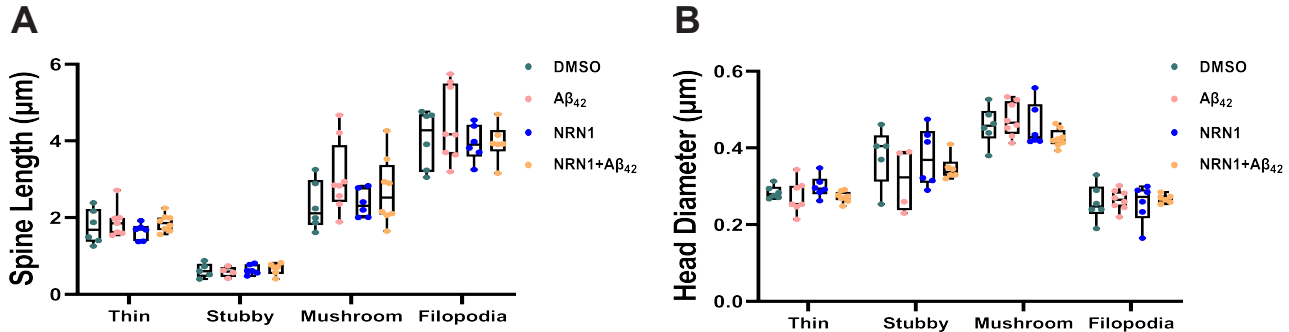

Supplement: Supplemental Figure S7 — Analysis of dendritic spine length and head diameter among thin, stubby, and mushroom spines, and filopodia.A, dendritic spine length of thin, stubby, or mushroom spines, and filopodia. B, dendritic spine head diameter of thin, stubby, or mushroom spines, and filopodia. Box plots represent median, 25th and 75th percentiles. Box hinges represent the interquartile range of the two middle quartiles with a group. Error bars are based on data points 1.5 times the interquartile range from the box hinge. [file mmc7.pdf]

Supplemental Figure 8

**A**

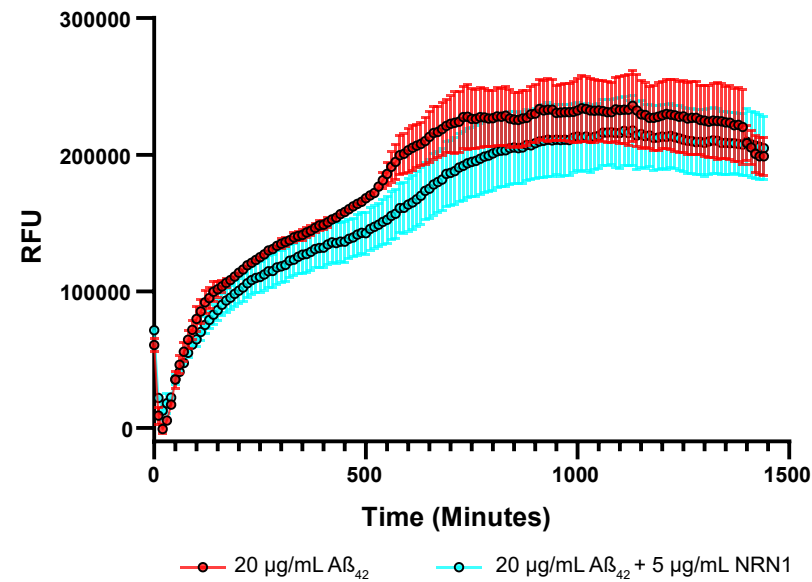

**B**

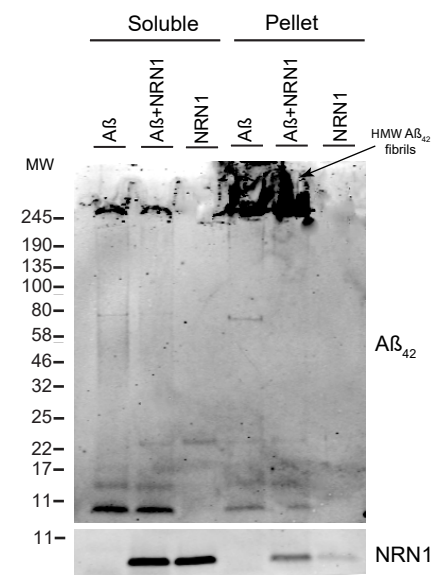

**C**

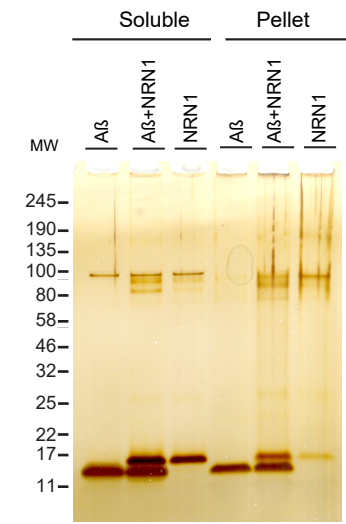

Supplement: Supplemental Figure S8 — Aggregation of Aβ in the presence or absence of NRN1.A, fibrillation curves of 20 μg/ml Aβ42 alone and 20 μg/ml Aβ42 + 5 μg/ml NRN1, thioflavin T (ThT) alone was recorded and subtracted as background. Relative fluorescent units (RFU) were recorded every 15 min for 20 h. Points are quadruplicate means ± SEM. B, Western blot of soluble and pelleted fractions of assay products probed for Aβ42 and NRN1. High molecular weight (HMW) fibrils are observed at the top of the gel. C, silver stain of soluble and pelleted fractions of assay products. [file mmc8.pdf]

Supplemental Figure 9

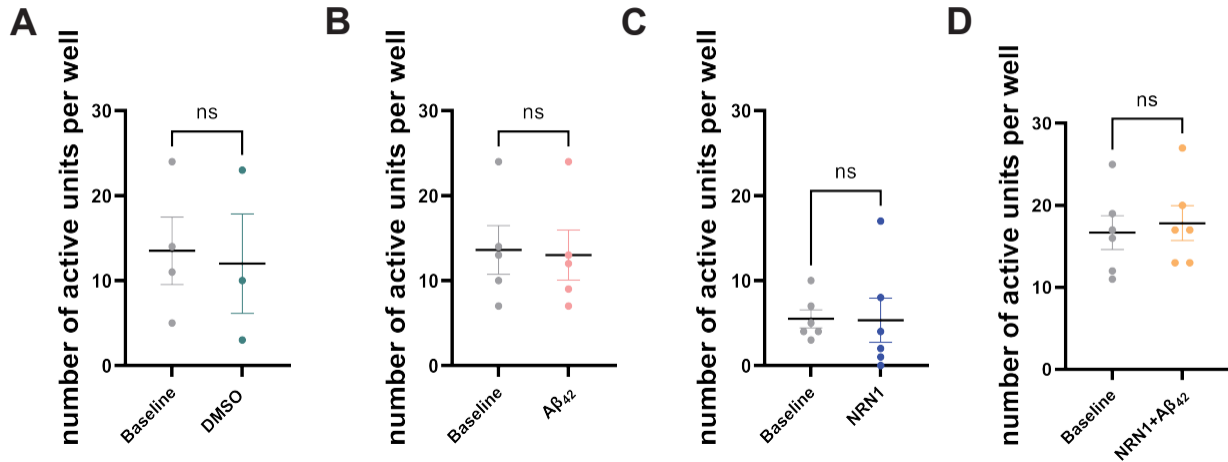

Supplement: Supplemental Figure S9 — Total number of active neurons per microelectrode array.A, the total number of active neurons per well at DIV 14 in hippocampal neurons treated with DMSO, compared to baseline (n = 3–4 wells with 64 electrodes/well, unpaired Student’s t test; p = 0.8339). Data are means + SEM. B, the total number of active neurons per well at DIV 14 in hippocampal neurons treated with 500 nM Aβ42, compared to baseline (n = 5 wells with 64 electrodes/well, unpaired Student’s t test; p = 0.8878). Data are means + SEM. C, the total number of active neurons per well at DIV 14 in hippocampal neurons treated with 150 ng/ml NRN1, compared to baseline (n = 6 wells with 64 electrodes/well, unpaired Student’s t test; p = 0.9539). Data are means + SEM. D, the total number of active neurons per well at DIV 14 in hippocampal neurons treated with 150 ng/ml NRN1 and 500 nM Aβ42, compared to baseline (n = 6 wells with 64 electrodes/well, unpaired Student’s t test; p = 0.7035). Data are means + SEM. [file mmc9.pdf]

Supplemental Figure 10

A

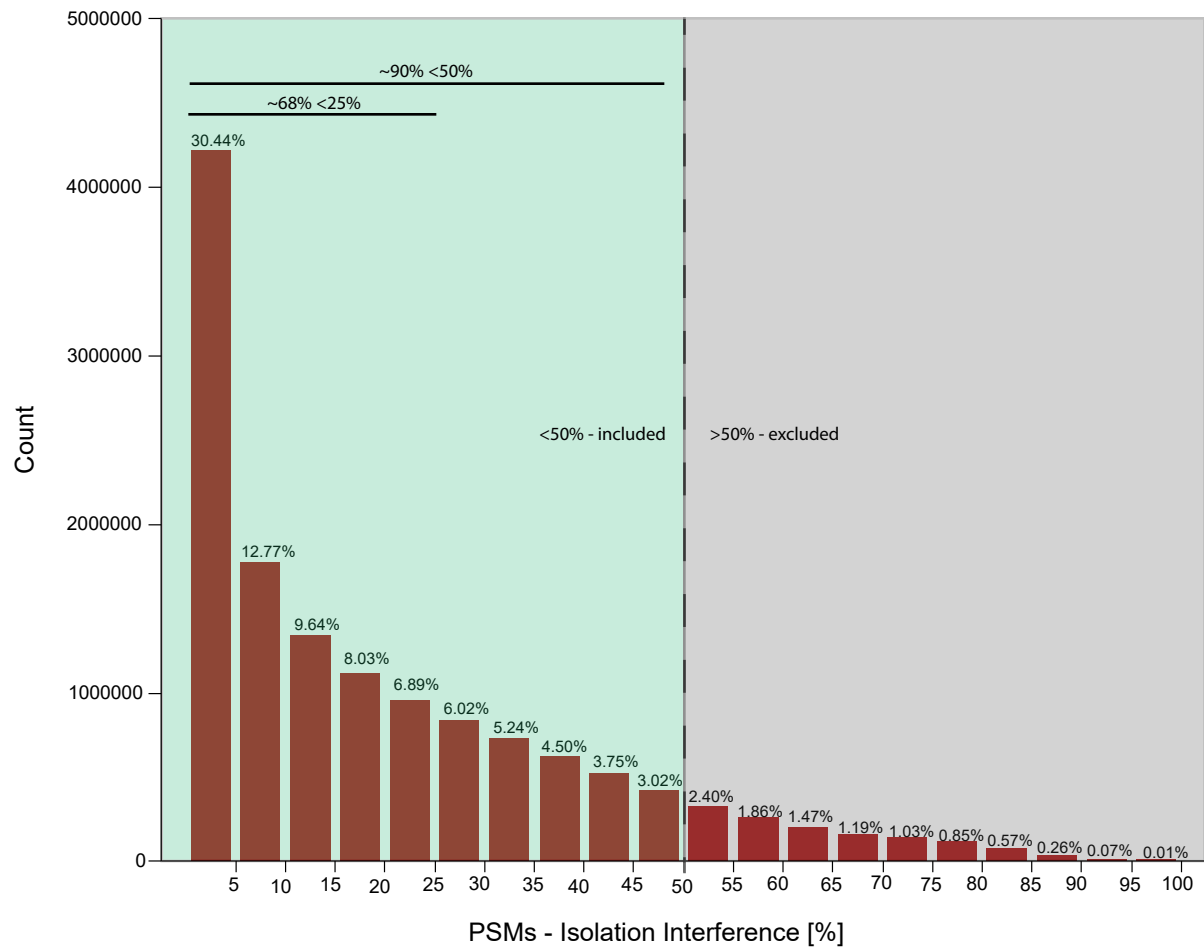

Supplement: Supplemental Figure S10 — Isolation interference of peptide spectral matches.A, percent isolation interference of all peptide spectral matches identified in BA6 and BA37 brain regions. Only spectra with less than 50% interference were used for quantitation. For interference less than 50%, approximately 90% of all spectra fell within this range and approximately 68% had less than 25% interference. [file mmc10.pdf]
